# Metabolomic and antioxidant characterization of seven Egyptian and Saudi date syrups via GC-MS and UHPLC/MS with sensory bioactive insights

**DOI:** 10.1038/s41598-025-19541-2

**Published:** 2025-09-18

**Authors:** Rabab M. Abdou, Heba A. Fahmy, Amira R. Khattab, Mohamed A. Farag

**Affiliations:** 1 Pharmacognosy Department, Faculty of Pharmacy, Cairo University, Cairo, Egypt; 2 Pharmacognosy Department, Faculty of Pharmacy, Modern University for Technology & Information, Cairo, Egypt; 3 Pharmacognosy Department, College of Pharmacy, Arab Academy for Science, Technology and Maritime Transport, Alexandria, 1029 Egypt; 4 Graduate School in Alamein, Arab Academy for Science, Technology and Maritime Transport, Alamein, Egypt

**Keywords:** Date fruit, Phoenix dactylifera, Metabolomics, Antioxidant, GC-MS, UHPLC-MS, Drug discovery, Plant sciences, Chemistry

## Abstract

Date syrup is a valued ingredient in Middle Eastern cuisine and is widely used in the food industry for its sweetness and high nutritional value, prepared from fruits post-heat processing. The current study assesses the chemical composition of 7 commercial date syrup products (D1-D7) of different origins *via* a metabolomics approach using hyphenated UHPLC-MS and GC-MS techniques, followed by multivariate data analysis. Primary metabolites profiling *via* GC-MS assigned 36 peaks belonging to sugars, sugar alcohols, fatty acids/esters, alcohols, organic acids, and nitrogenous compounds. Sugars and sugar alcohols amounted for ca. 97.04%, dominated by mono-sugars. Aroma profiling using headspace coupled to GC-MS revealed 17 volatiles belonging mainly to furans, alcohols, and esters, with furans likely produced upon processing as a major class in most products at ca. 48%, except samples (D2 and D3) where alcohols predominated at ca. 64%. Classification of date products was more reliable using silylated metabolites than from the aroma dataset. UHPLC-MS analysis of date syrups identified 77 metabolites, of which 33 are reported herein for the first time in *Phoenix dactylifera*, including sugars, phenolic acids, flavonoids, lignans, and fatty acids, with the former being the most abundant. The antioxidant effect of date syrups was attributed to their relatively high total phenolic and flavonoid contents. This study provides, for the first time, detailed profiling of date syrups from various origins to elucidate their nutritional composition and potential health benefits.

**Supplementary Information:**

The online version contains supplementary material available at 10.1038/s41598-025-19541-2.

## Introduction

Dates, the fruits of the *Phoenix dactylifera* palm tree, have a long and well-documented history. This nutrient-dense fruit has long been a chief nutritive source, especially in the Middle East and North Africa^1^. More than 5000 date cultivars are currently cultivated globally^2^ with Egypt the chief date producer, proceeded by Saudi Arabia^3^. Dates are classified into soft, semidry, or dry types. The fruit ripens, passing through four main stages viz. kimri, khalal, rutab and finally tamr^4^.

Date fruit is mostly composed of carbohydrates to amount for ca. 70% of the fruit^5^. The three main sugars in date fruit are fructose, glucose, and sucrose to provide between 160 and 230 kcal/100 g ^6^. Date fruit presents likewise a source of minerals (potassium, selenium, magnesium, calcium, iron, manganese), vitamins (e.g., A, C, B1, B2, and B9), proteins, dietary fibers, fatty acids, and carotenoids (e.g., neoxanthin and *β*-carotene). With regards to phytonutrients in date fruit, flavonoids, phenolic acids, anthocyanidins, sterols, procyanidins, tannins, and terpenoids are the major classes^2^. Aroma components contributing to fruit aroma include hydrocarbons, ketones, aldehydes, alcohols, esters, and fatty acids^4,7^. Date fruit’s chemical makeup varies depending on factors like growing environment, postharvest conditions, cultivar, and ripening stage. Sugars increase between the kimri and tamr stages^2^whereas sucrose turns into inverted sugar during the rutab stage, and because of the high activity of invertase, fructose peaks in tamr^8^. Compared to sugars, protein and lipid concentrations decrease upon ripening^7^. It should be noted that sample size plays a critical role in metabolomic and chemometric studies outcomes, as a limited number of samples may reduce the ability to detect patterns related to origin or other factors, and conclusions should therefore be drawn with caution.

Date fruit exhibits several health benefits e.g., antioxidant, immune-modulating, anti-inflammatory, anti-diabetic potential, cholesterol lowering, anti-tumor, nephro-protective, hepatoprotective, and cardiovascular disease-preventive potential. It can also promote the development of a healthy gut microbiota functioning as prebiotic, aside from its antibacterial, antiviral, and anti-fungal properties^9^.

Aside from date fruit consumption as whole fruit, several byproducts are very popular owing to their longer shelf life and culinary uses. Among date fruit major byproducts^5^date syrup, the “Dibs” or “Rub”, has been widely used for centuries as a natural sweetener, especially in North Africa and the Middle East. It is a favored substitute for honey and maple syrup and can be found in many recipes for dressings and marinades^10^. Also, it is widely used as a sweetener and flavoring in beverages, confections, baked goods, and ice cream. Date juice is usually extracted by boiling the dates in water, filtration, and then concentration. For juice clarification, several methods are employed including enzymatic hydrolysis, *via* cellulase and pectinase, or precipitation of colloids, centrifugation or filtration, color removal *via* ion exchange or activated carbon, and foaming to get rid of high molecular weight compounds^8^. Cellulase and pectinase are used to boost the extraction yield, lower sugars, soluble dry matter, and titrable acidity^11^. Such processing conditions are associated with date changes in fruit chemical composition. Numerous studies have demonstrated that heat-processed fruits and vegetables differ in health attributes from their fresh counterparts due to the thermal instability of many bioactive substances^12^. In contrast, thermal processing of certain vegetables and fruits was found to improve their nutritive value and health benefits^13,14^ owing to various underlying chemical changes e.g., Maillard reaction between free amino acids and reducing sugar, caramelization of sugars, and partial polyphenolic oxidation^15^. The dates syrup is rich in sugars (ca.71% mainly fructose and glucose), phenolics, carotenoids, flavonoids, minerals, with several effects i.e., antioxidant^15,16^antibacterial^17^and radioprotective activity^18^.

We have previously reported on date fruits from *cvs.* grown in Egypt and Saudi Arabia targeting its primary and secondary metabolites^19,20^. The current study sets out to further investigate chemical makeup of commercial date syrup of different origins in Egypt and Saudi Arabia as major producers of date syrup *via* a metabolomics approach based on the previously reported hyphenated UHPLC-MS and GC-MS techniques, to identify whether differences exist in these products, and how much in comparison to well-characterized date fruit chemical composition. Such comprehensive coverage of metabolites included primary and secondary metabolism among the different syrup products being subjected to disparities e.g., cultivars, post-harvest factors, and mostly processing conditions can aid in setting future quality control measures for this food product. In this study, gas chromatography (GC) was employed for profiling nutrients and aroma composition, whereas liquid chromatography (LC) targeted secondary bioactives i.e., phenolics, both coupled to mass spectroscopy (MS)^21^. To aid in the assessment of metabolite heterogeneity among syrup products, MS datasets were modeled using chemometric analyses exemplified by principal component analysis (PCA), hierarchical cluster analysis (HCA), and orthogonal partial least squares discriminant analysis (OPLS-DA). Further, in vitro antioxidant assay was performed to assess whether chemical differences are reflected on biological effects^6^.

## Materials and methods

### Date syrup products origin and Preparation for GC-MS and UHPLC-MS analysis

Five Egyptian and two Saudi Arabian date syrup products were provided from their industrial sources, with details listed in Table S1. The syrup products were lyophilized overnight to remove water. Three technical replicates were assessed for each product.

### Chemicals and fibers

The chemicals utilized were of analytical grade. Water, methanol, acetonitrile, and formic acid (≥ 95.0%, FA) were all of LC-MS grade and provided by Merck (Darmstadt, Germany). 1,1-Diphenyl-2-picrylhydrazyl (DPPH, about 90%), N-methyl-N-(trimethylsilyl)trifluoroacetamide (MSTFA), and 6-hydroxy-2,5,7,8-tetramethylchroman-2-carboxylic acid (Trolox)were supplied by Sigma (St. Loui, MO, USA). SPME fibres (StableFlex) coated with either polydimethylsiloxane (PDMS) or divinylbenzene/carboxen/polydimethylsiloxane (DVB/CAR/PDMS 50/30 µm) were acquired from Supelco (Oakville, ON, Canada) and were conditioned at 250 °C for five min as recommended by the supplier.

### GC-MS analysis of silylated primary metabolites

100 µL of the 50% aqueous extract (made by extracting 100 mg freeze-dried date syrup) with 50% MeOH (5 ml) for 3 h was frequent sonication over an ultrasonic bath (Branson Ultrasonic Corporation, Danbury, CT, USA). Hydroalcoholic extract was concentrated under nitrogen stream until it was completely dried to analyze primary metabolites (amino acids, organic acids, and sugars). Before GC-MS analysis, 150 µL of N-methyl-N-(trimethylsilyl)-trifluoroacetamide (MSTFA) was added and then incubated for 45 min at 60 ◦C for derivatization. Chromatography was performed on 1 µL of silylated products using an Rtx-5MS (30 m in length, 0.25 mm ID, and 0.25 μm film) column^22^. GC-MS conditions were in accordance with^23^and injections were made at a split mode (1:15). For every product, three technical replicates were examined. Identification of GC-MS peaks was made using AMDIS software (https://www.amdis.net), peaks were deconvoluted and then identified by mass spectra and retention indices (RI) matching to the NIST017 database. Relative quantification was expressed for each peak as a percentage to total peak areas^24^ .

### Headspace volatile analysis coupled to GC-MS

Date syrup volatiles were extracted using Headspace Solid-Phase Microextraction (HS-SPME) and subjected to GC-MS analysis from three technical replicates of each date syrup product. HS-SPME volatile analysis was carried out in accordance with our earlier report with minor changes^25^. Twenty milliliter screw-capped vials containing 3 g of the fresh syrup spiked with 10 µg hexenyl acetate as an internal standard were exposed to SPME fiber, which was manually inserted above the sample. Vials were placed in an oven kept at 50 °C for 20 min. After incubation time, the fiber was pulled back inside the needle and inserted into the GC injection port. GC-MS analysis and volatiles identification followed the conditions reported in ^25^.

### UHPLC-ESI-qTOF-MS/MS analysis of secondary metabolites

A mixture of date fruit syrups residue left after lyophilization (10 mg, each) was subjected to fractionation on a Diaion HP-20 column (15 cm×1 cm). Elution was performed using distilled water (50 ml) and methanol (50 ml), respectively. The pooled methanol fractions were collected, evaporated under reduced pressure and the residue was placed in a vial for LC-MS analysis. The dried pellet was redissolved in 100% methanol containing umbelliferone at 10 ug/ml as internal standard and filtered using a 22 μm filter before LC/MS analysis. Every sample (2 µL) was introduced into a Waters ACQUITY I-Class UHPLC system, comprising FL Sample Manager, Binary Solvent Module, and UHPLC eLambda 800 nm (wavelength 190–600 nm with resolution 1.2 nm), using partial injection mode. The samples were separated at a flow rate of 300 µL/min at 55 ◦C using a C18 column (Waters ACQUITY UHPLC BEH,50 mm × 2.1 mm internal diameter with 1.7 μm particle size, Waters GmbH, Eschborn, Germany). Water (A) and CH_3_CN (B) with 0.1% formic acid (an additive for LC-MS, LiChropur^®^, Merck) were the eluting solvents. (A) was obtained from BarnsteadTM GenPureTM, Thermo ScientificTM, and (B) Chromasolv™, for LC-MS, Honeywell Riedel de Ha¨en™). A gradient of optimum elusion was used for the chromatographic separation, with solvent B starting at 3% (isocratic, 1 min), raising to 95% B for 7 min, subsequently at 95% B for 3 min, and finally re-equilibrating the column at 3% B for 2.5 min. A hybrid qTOF mass spectrometer (Sciex TripleTOF 6600 LC-MS System, AB Sciex, Darmstadt, Germany) operating in negative ion mode was online infused with the column effluents. The source temperature was set to 450 ◦C, the voltage of ion spray to − 4500 V, and the nebuliser to 85, drying to 70, and curtain gases to 55 psig, The MS acquisition in the TOF-scan mode with an accumulation time of 100 ms, within 50–1500 m/z range. In the information-dependent acquisition mode (IDA), the MS2 experiments were completed with a 50-minute accumulation time with collision potential (CE) of − 40 V, collision energy spread (CES) 10 V, and declustering potential (DP) − 35 V. The mass tolerance of 25 ppm, intensity ~ more than 100, and exclude isotope window 4 Da were also met. The collision activation dissociation (CAD) gas used in this experiment was nitrogen.

### UHPL-MS data processing and peaks identification

PeakView software version 2.1 (AB Sciex) was used to visualize and process raw UHPLC-MS files. ProteoWizard 3.0 MSConvert was then used to convert the files to mzXML format, which was then processed using Mzmine 2.53 software. Mass ion peaks were separated using a centroid mass detector threshold succeeded by a chromatogram builder and deconvolution via baseline cut-off algorithm. The gap-filling peak finder was used to identify missing peaks after the isolated peaks were deisotoped using the isotopic peak grouper. The *m/z* tolerance of 0.001 m/z or 5.0 ppm, the retention time tolerance of 0.2 absolute (min), and the minimum standard intensity of 5 × 103 were the parameters set for the isotopic peaks’ grouper and gap-filling. Each peak list was aligned using the join aligner, and the resulting aligned peak list was exported as a CSV file (Excel 2016, Microsoft^®^, Redmond, WA, USA). This allowed each feature in every sample to have its feature ID number, *m/z*, Rt, and peak intensity provided. Peaks were identified based on molecular formula prediction, fragment MS spectra in comparison with previous reports on date metabolites.

### Multivariate data analysis of GC-MS and UHPLC/MS datasets

GC-MS and UHPLC-MS datasets were modeled using SIMCA-P version 13.0 (Umetrics, Umea, Sweden), with all variables scaled to Pareto variance and mean-centered. To determine the ideal number of principal components required for data modelling in PCA and HCA, a seven-fold cross-validation approach was employed. The distance to the model (DModX) test was then utilized to confirm the existence of outliers. To further compare syrup products, OPLS-DA was used. The developed OPLSDA models were validated using permutation tests and ANOVA of cross-validated residuals. The derived S-plot, which was designated with covariance (p) and correlation (pcor) along with the variable importance in the projection (VIP), was then analyzed to identify the metabolite markers. The bioactivities and the identified phytochemicals were correlated using partial least squares (PLS) analysis. The main contributing variables were then identified using the VIP approach. Regression analysis and permutation tests were used to achieve PLS validation. Using RStudio version 2022.07.1 (Rstudio, Boston, MA, USA), a correlogram plot was produced. The following guidelines were used to define the size of correlation (r) between the bioactivities and the identified metabolites: There are four types of correlation: negligible (*r* < 0.3), low (*r* = 0.3–0.5), moderate (*r* = 0.5–0.7), high (*r* = 0.7–0.9), and very strong (*r* = 0.9–1.0) ^26^.

### Total phenolics and total flavonoids content

Folin–Ciocâlteu method^27^ was used to determine the total phenolic content using gallic acid as a reference standard and the results were expressed as gallic acid equivalent (GAE) in mg/g sample. The total flavonoid content was measured according to the AlCl_3_ method^28^ using quercetin as a reference standard, and results were expressed as quercetin equivalent (QE) in mg/g extract. UV-visible microplate spectrophotometer, Shimadza UV-1650 PC, was used to measure the absorbance.

### In vitro DPPH radical scavenging assay

The free radical scavenging activity of the date syrup samples were determined following the method described by^29^ with slight modifications. In summary, a 96-well plate was filled with 20 µL of the sample, which consisted of lyophilized product of date syrup methanol extracts diluted serially to 2000 µg/mL. Next, 180 µL DPPH in methanol (100 µM) was added to the mixture. The solution was left in the dark for 20 min, and the intensity of DPPH color was measured at 540 nm. Gallic acid was used as a standard reference and results were expressed as IC_50_ using microplate reader (Infinite F50, Tecan, Switzerland) to measure the absorbance. The absorbance was recorded using UV-visible microplate spectrophotometer, Shimadza UV-1650 PC. The following equation describes the data representation: In % Scavenging, Ab is the blank absorbance of and As is the sample absorbance, calculated as [(Ab − As) /Ab)]X 100.

## Results and discussion

The Study’s main goal was to assess date syrup metabolome in the context to its nutritive, aroma, and secondary metabolites *via* a multiplex approach of GC-MS and UHPLC-MS techniques, and visualized using chemometrics, to classify among different syrup products (D1-D7), see Table S1.

### Primary metabolites profiling of date syrup via GC-MS analysis (Post-Silylation)

**Fig. 1:**
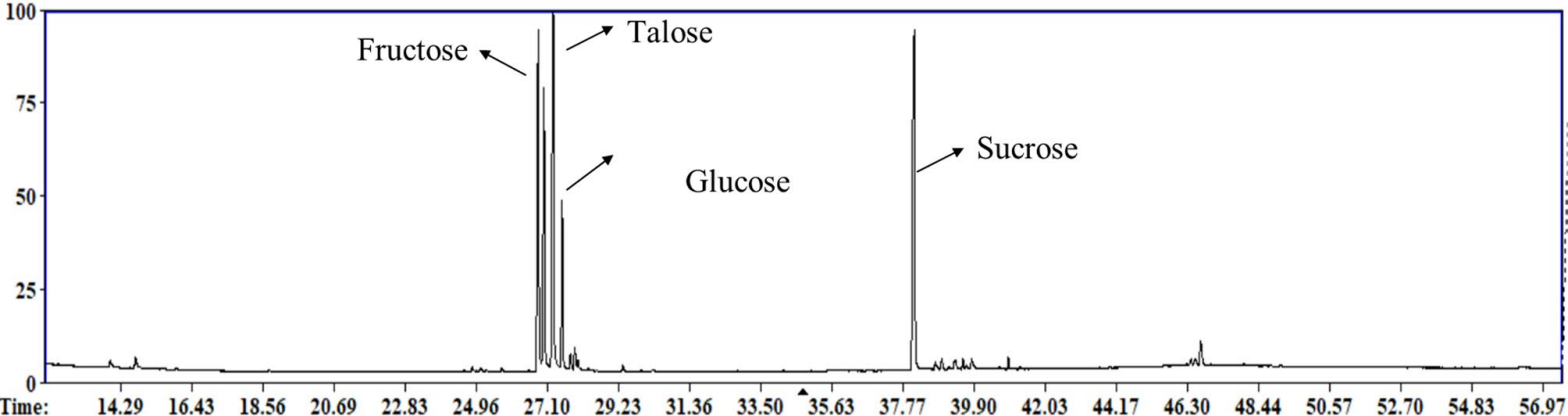
Representative GC-MS chromatogram of silylated metabolites in dates syrup samples.

**Fig. 2:**
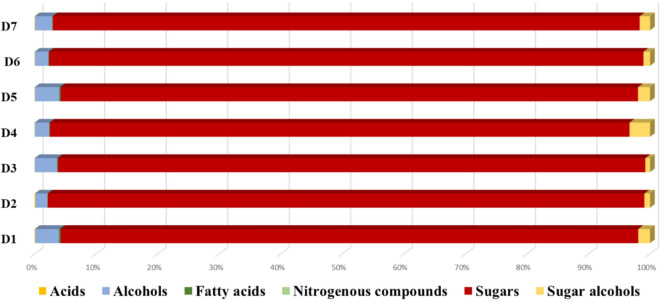
A bar chart illustrating the percentage of major metabolite classes in date syrup samples.

Post-silylation GC-MS analysis was employed to assess differences in primary metabolites among date syrup products mediating their nutritive value and sensory attributes. Seven Egyptian and Saudi Arabian commercial date syrup products were examined, and their codes are explained in (Table S1). Three separate technical specimens were used to evaluate the technical variance for each date preparation (Fig. 1). A total of 36 peaks (Table 1) were identified, including sugars (mono- and disaccharides), sugar alcohols, fatty acids/esters, alcohols, organic acids, and nitrogenous compounds. Figure 2 shows the percentage of major metabolite classes in date syrup samples. As expected, sugars amounted for the major class and mirroring metabolite composition in date fruits^19^.

**Table 1:** Relative percentage of silylated metabolites in dated syrups analyzed via GC–MS, *n* = 3. For codes, refer to table S1. Results are represented as an average of 3 independent replicates ± std. Deviation.

Peak	Average Rt (min)	RI	Metabolite name	Class	D1	D2	D3	D4	D5	D6	D7
1	16.252	1362	Fumaric acid (2TMS)	acid	0.02 ± 0.00	-	0.01 ± 0.00	0.01 ± 0.00	0.01 ± 0.00	-	0.01 ± 0.00
2	25.74	1855	Citric acid (4TMS)	acid	0.07 ± 0.02	0.12 ± 0.05	0.02 ± 0.01	0.03 ± 0.01	0.05 ± 0.00	0.04 ± 0.01	0.06 ± 0.02
Total acids					0.09	0.12	0.03	0.05	0.05	0.05	0.07
3	13.992	1258	Diethylene glycol (2TMS)	alcohol	3.63 ± 2.31	1.88 ± 1.38	3.56 ± 1.73	2.10 ± 1.38	3.80 ± 2.23	2.09 ± 1.64	2.68 ± 1.50
4	14.738	1295	Glycerol (3TMS)	alcohol	0.24 ± 0.08	0.07 ± 0.03	0.06 ± 0.06	0.24 ± 0.04	0.17 ± 0.01	0.10 ± 0.01	0.13 ± 0.01
Total alcohols					3.87	1.95	3.63	2.34	3.97	2.20	2.81
5	29.085	2059	Palmitic acid, TMS	fatty acid	0.09 ± 0.06	0.01 ± 0.01	0.04 ± 0.03	0.04 ± 0.01	0.09 ± 0.09	0.03 ± 0.02	0.03 ± 0.01
6	31.542	2227	Octadecadienoic acid TMS	fatty acid	0.04 ± 0.03	0.01 ± 0.00	0.01 ± 0.01	0.02 ± 0.01	0.01 ± 0.01	0.01 ± 0.00	0.01 ± 0.01
7	32.051	2259	Octadecanoic acid (TMS)	fatty acid	0.08 ± 0.07	0.02 ± 0.00	0.09 ± 0.08	0.03 ± 0.01	0.09 ± 0.09	0.05 ± 0.01	0.02 ± 0.01
8	36.773	2592	Palmitic acid, 2-(1-octadecenyloxy)ethyl ester, (E)-	fatty acid	0.02 ± 0.04	-	-	-	0.03 ± 0.04	-	-
Total fatty acids					0.24	0.04	0.14	0.09	0.21	0.08	0.07
9	20.136	1620	Serotonin (5TMS)	nitrogenous	0.01 ± 0.00	0.02 ± 0.00	0.01 ± 0.00	0.01 ± 0.00	-	0.01 ± 0.00	0.01 ± 0.00
Total nitrogenous compounds					0.01	0.02	0.01	0.01	0.00	0.01	0.01
10	22.304	1710	D-Xylulose (4 TMS)	sugar	0.01 ± 0.00	-	0.39 ± 0.51	0.01 ± 0.01	0.02 ± 0.01	0.10 ± 0.04	0.01 ± 0.00
11	22.773	1720	Maltotriose (11 TMS)	sugar	0.04 ± 0.00	0.03 ± 0.01	0.04 ± 0.03	0.05 ± 0.00	0.06 ± 0.01	0.02 ± 0.00	0.04 ± 0.02
12	24.621	1789	Unknown	sugar	0.37 ± 0.05	0.13 ± 0.02	0.08 ± 0.02	0.86 ± 0.05	0.97 ± 0.10	0.16 ± 0.01	0.30 ± 0.06
13	24.866	1802	Ribofuranose (4TMS)	sugar	0.76 ± 0.06	0.31 ± 0.09	0.44 ± 0.12	0.72 ± 0.08	0.59 ± 0.11	0.44 ± 0.02	0.66 ± 0.09
14	26.561	1903	Tagatose (5TMS)	sugar	0.01 ± 0.00	-	-	0.01 ± 0.00	-	-	-
15	26.86	1920	Fructose (5TMS)	sugar	26.78 ± 0.55	17.18 ± 1.35	12.38 ± 1.59	28.06 ± 0.05	28.22 ± 1.27	14.15 ± 5.76	24.99 ± 3.69
16	27.022	1931	Fructose (5TMS) isomer	sugar	20.02 ± 0.51	11.89 ± 0.91	9.28 ± 0.94	19.99 ± 0.35	20.22 ± 0.58	12.34 ± 0.81	18.09 ± 2.47
17	27.182	1941	D-Allose (5TMS)	sugar	-	0.01 ± 0.01	7.58 ± 13.13	0.00 ± 0.01	0.01 ± 0.01	7.21 ± 12.48	8.15 ± 14.12
18	27.305	1949	Talose (5TMS)	sugar	31.68 ± 0.44	19.42 ± 1.19	25.29 ± 1.78	30.55 ± 0.78	29.75 ± 0.61	23.44 ± 1.81	29.33 ± 4.07
19	27.568	1965	Glucose (5TMS)	sugar	10.96 ± 0.27	6.47 ± 0.53	8.22 ± 0.91	10.56 ± 0.20	10.23 ± 0.13	7.77 ± 0.58	10.05 ± 1.47
20	28.587	2029	D-Mannose (5TMS)	sugar	0.06 ± 0.02	0.04 ± 0.01	0.07 ± 0.01	0.14 ± 0.05	0.08 ± 0.01	0.05 ± 0.01	0.07 ± 0.03
21	29.376	2079	Glucose (5TMS) isomer	sugar	0.60 ± 0.04	0.24 ± 0.08	0.29 ± 0.04	0.52 ± 0.06	0.45 ± 0.09	0.34 ± 0.02	0.52 ± 0.06
22	38.091	2682	Sucrose (8TMS)	sugar	0.39 ± 0.19	28.90 ± 3.13	0.18 ± 0.10	0.19 ± 0.02	0.49 ± 0.03	0.06 ± 0.02	0.67 ± 0.12
23	38.24	2695	Cellobiose (8TMS)	sugar	0.18 ± 0.29	9.75 ± 8.39	0.96 ± 0.23	0.01 ± 0.01	0.03 ± 0.02	1.05 ± 0.15	0.02 ± 0.00
24	38.954	2743	Turanose (8TMS)	sugar	0.56 ± 0.05	0.40 ± 0.08	0.13 ± 0.03	0.66 ± 0.03	0.70 ± 0.03	0.15 ± 0.02	0.49 ± 0.09
25	39.243	2765	Palatinose (7TMS)	sugar	0.05 ± 0.07	0.00 ± 0.00	0.01 ± 0.00	0.01 ± 0.00	0.01 ± 0.00	0.01 ± 0.00	0.00 ± 0.00
26	39.367	2774	Maltose (8TMS)	sugar	0.27 ± 0.24	0.31 ± 0.16	14.99 ± 2.72	0.58 ± 0.02	0.50 ± 0.01	14.14 ± 1.38	0.81 ± 0.11
27	39.589	2787	Sucrose (8TMS) isomer	sugar	0.58 ± 0.05	0.37 ± 0.08	0.10 ± 0.02	0.59 ± 0.03	0.64 ± 0.00	0.16 ± 0.03	0.38 ± 0.06
28	39.686	2791	Gentiobiose (8TMS)	sugar	0.17 ± 0.01	0.09 ± 0.04	5.90 ± 1.16	0.16 ± 0.01	0.16 ± 0.00	5.65 ± 0.53	0.26 ± 0.04
29	40.01	2797	Trehalose (8TMS)	sugar	0.02 ± 0.01	0.10 ± 0.15	0.02 ± 0.01	0.03 ± 0.01	0.10 ± 0.07	0.02 ± 0.00	0.03 ± 0.01
30	40.368	2843	2-α-Mannobiose (8TMS)	sugar	0.05 ± 0.00	0.02 ± 0.01	0.02 ± 0.01	0.08 ± 0.02	0.08 ± 0.03	0.04 ± 0.01	0.04 ± 0.00
31	48.751	3430	Maltose (8TMS) isomer	sugar	-	0.01 ± 0.02	7.03 ± 1.54	-	-	7.38 ± 0.86	0.01 ± 0.01
32	49.213	3464	Maltose (8TMS) isomer	sugar	-	-	1.94 ± 0.51	-	0.00 ± 0.00	1.79 ± 0.26	0.00 ± 0.00
33	28.995	2054	Gluconic acid (5TMS)	sugar	0.31 ± 0.04	0.02 ± 0.01	0.11 ± 0.03	0.34 ± 0.01	0.40 ± 0.02	0.06 ± 0.01	0.40 ± 0.07
34	46.759	3290	Sucrose isomer (8TMS) isomer	sugar	0.01 ± 0.00	1.24 ± 0.29	-	0.04 ± 0.00	0.08 ± 0.06	0.07 ± 0.12	0.02 ± 0.00
Total sugars					93.87	96.94	95.44	94.18	93.81	96.60	95.36
35	30.289	2142	Inositol (6TMS)	sugar alcohol	0.14 ± 0.07	0.06 ± 0.02	0.08 ± 0.01	0.34 ± 0.02	0.16 ± 0.02	0.06 ± 0.03	0.11 ± 0.05
36	27.938	1987	D-Mannitol (6TMS)	sugar alcohol	1.77 ± 0.05	0.86 ± 0.15	0.68 ± 0.12	3.00 ± 0.07	1.79 ± 0.05	1.01 ± 0.10	1.57 ± 0.26
Total sugar alcohols					1.92	0.92	0.76	3.34	1.95	1.07	1.68
					100.00	100.00	100.00	100.00	100.00	100.00	100.00

### Sugars

Sugars were detected as the most dominant primary metabolite class as expected considering that date fruit is a chief carbohydrate source^30^. Sugars were represented by 27 peaks of sugars and sugar alcohols amounting to 97.04%, ensuring a good preservative effect on the date syrup products and shelf life^31^and justifying their use as a natural sweetener in different recipes.

Mono-sugars predominated all syrup products detected at 90–94%, posing them as instant sources of energy^32^ except one sample obtained from Saudi Arabia (D2) and two samples from Egypt (D3 & D6) which showed lower levels at 56–66%. Next to mono-sugars, di-sugars were most abundant in D2, D3, and D6 at 30–40%, compared to others in which it reached ca. 2.52%. Such higher levels of mono-sugars in the products, coupled with lower levels of di-sugars compared to D2, D3 & D6, may suggest that they were subjected to more extensive hydrolysis using higher temperature or prolonged time during processing. Conversely, the higher di-sugars content in D2, D3, and D6 may indicate possible addition of sucrose to improve taste or shelf life.

The most abundant mono-sugars included fructose, talose, and glucose, and following our previous report on date fruits^19^. Fructose (peaks 15, 16) was detected as the main sugar at considerably higher levels of 43–48% in all products except D2, D3, and D6 imparting a notably sweet taste. Talose (peak 18) was the second major sugar detected at a range of (19.42–31.68%). While glucose detected in peaks 19 & 21 ranged from ca. 7% in D2, D3 and D6 samples to ca.11% in the remaining samples.

Allose (peak17), the non-caloric sweetener was found to be exceptionally high in D3, D6, and D7 compared with other date syrup products at 5.48–8.15%, respectively. Compared to mono-sugars, the most abundant di-sugar was found to be sucrose at the highest level in D2 (29.28%) compared to ca. 1% or less in the other products. The sugar profile poses D3 and 6 as the products with the best-balanced sweetness due to their higher maltose and allose coupled with lower fructose levels.

Such a high concentration of simple sugars poses date syrup as an instant energy source is similar to date fruit, albeit, is deprived of the high fiber blessing of the fruits^33^ that slows down sugar absorption^34^. The observed variation in sugar contents among the examined syrup could be explained based on dates’ type, cultivar, degree of maturation, processing conditions, and\or addition of sweeteners during preparation. As soft date cultivars (e.g., Saidy and Barhi) are predominated with fructose and glucose with traces of sucrose, whereas dry cultivars (e.g., Deglet Beidha and Noor) contain higher sucrose levels, and others (e.g., Halawy, Khadrawy, Sayer, Zahidi) contain mixed sugars^35^. The ratio of fructose to sucrose differs widely among cvs. ranging from 3.3 to 6.5 and reaching as low as ca. 0.3 in some dry cultivars^36^. Considering the stage of maturity, at the early Rutab stage, sucrose constitutes ca. 60% of the dry weight of date, while fructose increases during other stages and reaches its maximum in Tamr stage due to the high invertase activity. Considering that date syrup is mainly prepared in the Tamr and Rutab stages, fructose was the main detected sugar as expected in most of the products. D2 exceptionally high sucrose level could be either due to the date cultivar, stage of maturity or adulteration, hence at least *cvs* and stage of maturity of the utilized date fruits should be stated on the product label. Whether these date syrups were subjected to pectinase treatment if prepared from high fiber *cvs.* type^8^ has yet to be determined.

In contrast to free sugars, sugar alcohols were detected at lower levels in all syrup products represented by mannitol (peak 36) and inositol (peak 35) ranging from 0.8 to 3.3%. Mannitol, the low glycemic index sweetener, was detected at 0.68-3%. While inositol was previously detected in dates^19^.

Dates have been traditionally used to treat diabetes mainly because of its high fructose level, in addition to other phytochemicals such as anthocyanins and flavones^6^. Nearly all examined products appear suitable for diabetic patients due to their high fructose and mannitol content compared to glucose and sucrose except D2, which has yet to be confirmed by measuring the increase in post-prandial sugar levels post-consumption in animals or ideally human trials.

### Fatty acids/esters

Fatty acids/esters were detected at very low levels in date syrup with the highest content in D1 and D5 products obtained from Egypt at 0.2%. Both saturated fatty acids, e.g., palmitic acid (peak 5), stearic acid (peak 7), and monounsaturated fatty acids (MUFA) e.g., octadecadienoic acid (peak 6), as well as palmitic acid were annotated at trace levels of less than 0.1% each. Such low levels of fatty acids mirror that in fruits, especially in the tamr stage^37^and suggestive that most of these syrups were prepared from fruits at tamr ripening stage. Several saturated fatty acids, MUFA, and polyunsaturated fatty acids (PUFA) previously detected in the Egyptian dates fruit were absent or detected in trace levels in the syrup including palmitic, oleic, linoleic and linolenic acids^19^ likely attributed to the processing conditions which start by extraction with water, leaving behind most of non-polar metabolites^8^.

### Organic acids/alcohols

Organic acids, represented by fumaric acid (peak 1) and citric acid (peak 2), were detected at trace levels (lower than 0.12%) in most date syrup, with D2 being the most enriched. Compared to date syrup, the percentage of organic acids in fruits is much higher, imparting a sour taste in fruits, especially the unripe fruits^19^and confirming that the syrup was prepared from fully mature fruits. On the other hand, alcohols were detected at higher levels in syrups (2.2–3.97%) represented by diethylene glycol (peak 3) and glycerol (peak 4).

### Multivariate PCA, HCA and OPLS-DA analyses of GC-MS silylated dataset

#### Unsupervised multivariate data analysis PCA and HCA of date syrup products

The comprehensive evaluation of primary metabolite heterogeneity among commercial date syrup products was further conducted using chemometric tools. This was done considering the notable number of peaks that were found as variables in 7 selected products, each of which is represented by three technical replicates, for a total of 21 date syrup products.

**Fig. 3:**
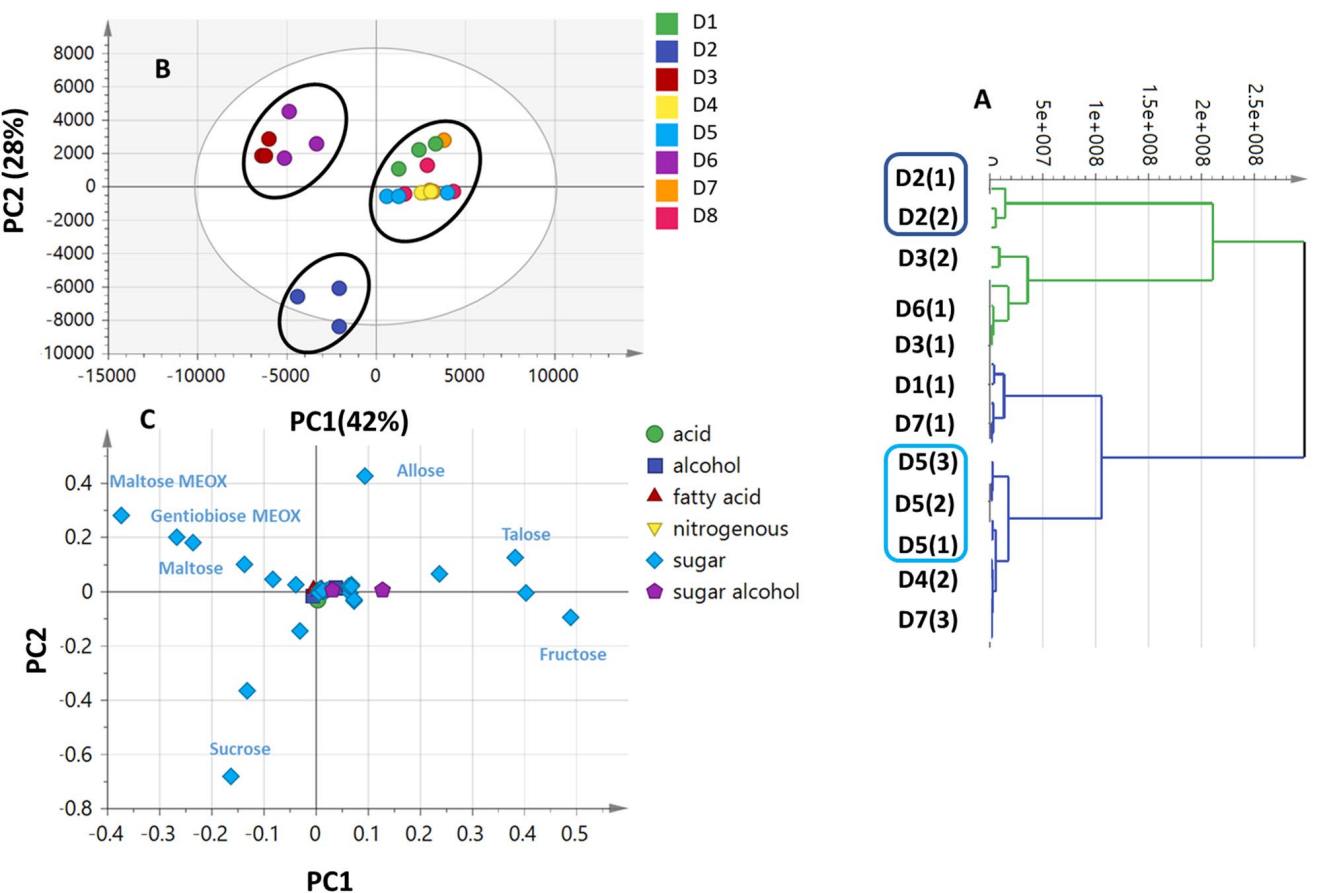
GC-MS based HCA and PCA of primary metabolites from date syrup samples (*n* = 3). (**A**) HCA plot. (**B**) Score plot of PC1 vs. PC2 scores. (**C**) Loading plot for PC1 & PC2 contributing metabolites and their assignments. The metabolome clusters are located at the distinct positions in two-dimensional space described by two vectors of principal component 1 (PC1) = 42% and PC2 = 28%.

The unsupervised HCA (Fig. 3A) depicted that D2, D3, and D6 were clustered together, while D1, D4, D5, and D7 were in another cluster. D2 individual clustering from all syrup products is attributed to its richness in sucrose (ca. 29%). D3 and D6 separate clustering can be linked to their richness in maltose, and gentiobiose as revealed from GC-MS analysis (Table 1), and suggestive for different sugar profiles among syrup preparations. PCA model prescribed by PC1 and PC2 accounting for 42% and 28% of the variance, respectively, (Fig. 3B) showed D2 as clearly segregated on the lower left side, whereas D3 and D6 were positioned in the upper left quadrant. Nonetheless, a clear overlap between the independent technical replicates was noted in the remaining products. Sugars (maltose, gentiobiose, sucrose, allose, talose, and fructose) accounted for specimens’ segregation as revealed from PCA loading plot. D2 was enriched in sucrose, versus the richness of D3 and D6 in di-sugars i.e., maltose and gentiobiose. D1, D4, D5 and D7 clustering was mainly associated with their richness in their mono-sugars (Fig. 3C). To improve separation, supervised OPLS-DA analysis was further used to reduce the variance between replicates for every sample.

#### Supervised multivariate data analysis OPLS-DA

In an effort to distinguish between the products’ independent replicates and to further identify metabolite markers responsible for segregation of D2, D3, and D6, as determined using unsupervised HCA and PCA (Fig. 3), supervised OPLS-DA (Fig. S1A) was carried out. D2, D3, and D6 were modeled in one class against different products *via* supervised OPLS-DA. The high prediction power was indicated by the developed model’s good sample separation, R2 (98.6%), and Q2 (96.5%) (Fig.S1). The segregation of D2, D3, and D6 from all other products was evident in OPLS-DA score plot. Di-sugars (viz. maltose, gentiobiose, sucrose, and cellobiose) were the primary discriminators of D2, D3, and D6 as revealed from OPLS-DA loading S-plot (Fig. S1B), and in accordance with GC-MS results (Table 1), posing such samples as better sources for sustained energy^32^. In contrast, mono-sugars viz. fructose and talose were responsible for segregation of D1, D4, D5 and D7 (Fig. S1B) posing them as better instant energy sources^32^and suggestive for di-versus mono-sugars abundance in date syrups. With a *p*-value of less than 0.05, the permutation test was used to validate the developed OPLS-DA model and confirm that it is statistically significant (Fig. S2). ANOVA was performed on the most discriminatory metabolites (Table S2) revealing significant differences in their content in the studies samples with a confidence level of 95% i.e. Fructose (5TMS) level was significantly higher in D1 and D5 samples, however, talose (5TMS) level was significantly higher in D1, D4 and D5 samples. D3 and D6 samples showed the highest significant levels of gentiobiose (8TMS), Maltose (8TMS) isomer at *P* ≤ 0.05.

### Aroma profiling of different date syrup products via SPME–GC–MS

**Fig. 4:**
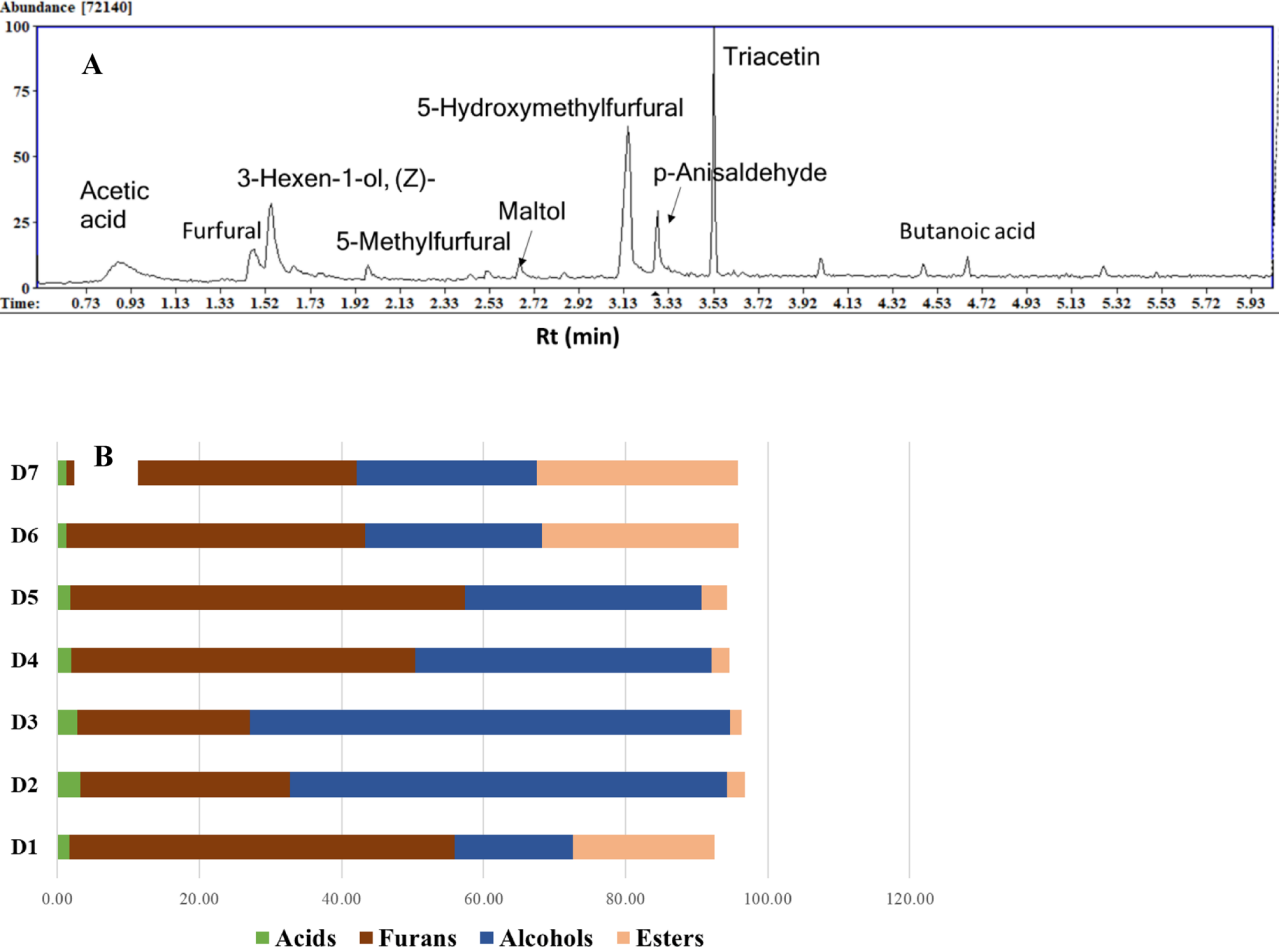
Representative GC/MS chromatogram of volatile metabolites in date syrup samples (**A**), bar chart illustrating the percentage of major volatile metabolite classes in date syrup samples (**B**).

Considering date syrup-rich aroma and to discern whether there is a unique aroma profile for certain syrup preparations versus others, and further how date syrup aroma compares to its date fruit, headspace–solid-phase micro-extraction (HS-SPME) was employed as a cold extraction technique for aroma collection^38^. Differences among commercial products were evident in terms of the quantity of volatile compounds rather than their composition. A representative chromatogram is shown in Fig. 4A. The major detected volatiles mainly belonged to three chemical classes, viz. furans, alcohols, and esters, along with five classes detected at trace levels viz. acids, hydrocarbons, aldehydes, pyranones, and pyrroles (Table S2, Fig. 4B). A total of 17 volatile compounds were detected, namely 5 alcohols (38.71%), 4 furans (42.15%), 2 acids (2%), 2 pyrroles (2.86%), in addition to an aldehyde, ester, hydrocarbon and pyranone (Table S2). The identified constituents represented ca. 87.68%of the total date syrup aroma. Furans amounted to 24.3–55.5% of syrup products’ aroma likely derived from sugar oxidation upon heating, followed by alcohols amounting to 16.6–67.7%. Furans were the most abundant class in all products except in D2 and D3 where alcohols predominated at 61.43 and 67.71%, respectively. Acids constituted 1.3–3.2% with the highest percentage in D2.

Furans were dominated by furfural and 5-hydroxymethylfurfural (5-HMF) to likely impart almond-like odor^39^ mixed with mild-sweet caramel aroma^40^. Furfural was detected in all products ranging from 14.7 to 22.5% in D1, except in D3 only (ca. 7%). Such hypothesis was confirmed from 5-HMF detected at 24-30.4%, except in D2 and D3 (ca. 9.8 and 16.4%, respectively) suggestive of either no thermal heat application in the latter products or their treatment to remove furans. Chiefly, Furfural and HMF are formed during concentration, and storage *via* the Maillard reaction due to the effect of heat on reducing sugars, and to serve as quality indicators for both preparation and storage conditions. Several approaches have been developed to minimize or remove 5-HMF from foods, e.g., UV irradiation, vacuum treatment, yeast fermentation, non-thermal processing, microwave heating^41^and gamma irradiation^42^. Compared to date syrup, furans in fruits were detected at low levels (1–3%) confirming that their formation is during processing and storage^43^.

Alcohols were dominated by 3-hexen-1-ol (peak 4), isopropyl alcohol (peak 3), and 4-penten-2-ol (peak 5). The most abundant alcohol was 3-hexen-1-ol imparting green aroma in green tea^44^with the highest amount in D3 and D2 (48–58%), while D1 showed the lowest level at *11.85*%. Moreover, isopropyl alcohol was detected only at higher levels in D2, D4, D5, and D3 (6–10%), which may impart a disagreeable aroma in these preparations^7^. D1, with its high 5-HMF and low isopropyl alcohol content, provides the best sensory attributes, having a sweet aroma opposite to D3. While the highest amount of 4-penten-2-ol, was detected in D6, D7, and D4 at 5–7%, versus 1–2% in remaining products. Although 3-hexen-1-ol and 4-penten-2-ol were not reported in date fruit aroma, their analog compounds viz. 2-hexenal, 3-hexenal, hexanal, 1-hexanol, 1,4-pentadien-3-ol, and isopentanol were detected^7,43^while isopropyl alcohol was previously reported as a component of date fruits aroma^45^. Such volatiles may also be derived either from lipid peroxidation or Maillard reactions of amino acids during syrup processing. Alcohols and furans exert antibacterial qualities that improve food safety and preservation^46,47^in addition to enhancing flavor. As previously reported, esters amount for major class in date fruits imparting fruity odor^37^. Likewise in syrup, an unknown ester was detected (peak 8) with the highest levels in D1, D6, and D7 (20–28. %).

The detected volatiles, especially alcohols, aldehydes, and esters, are likely to contribute the most to date syrup aroma profile. Compared with the rich aroma of date fruit revealed in our previous study^43^its composition greatly varied in the case of syrup. As observed, several compounds were not detected, especially terpene hydrocarbons, oxygenated monoterpenes, benzenoids, in addition to many esters, aldehydes, alcohols, and acids. While others appeared viz. furfurals, pyranones and pyrroles likely during thermal heating of date syrup. Compared to aroma of date palm fruit, syrup can vary based on more factors such as the type of date fruit, processing techniques, and storage conditions. Different processing methods utilized e.g., thermal, hydraulic pressure, microwave, ultrasound, and ohmic heating, greatly influence the produced syrup^33^and should be compared in the context of syrup metabolite profile to identify best methods in the preparation.

### Multivariate data analyses of date syrup products’ aroma profile

**Fig. 5:**
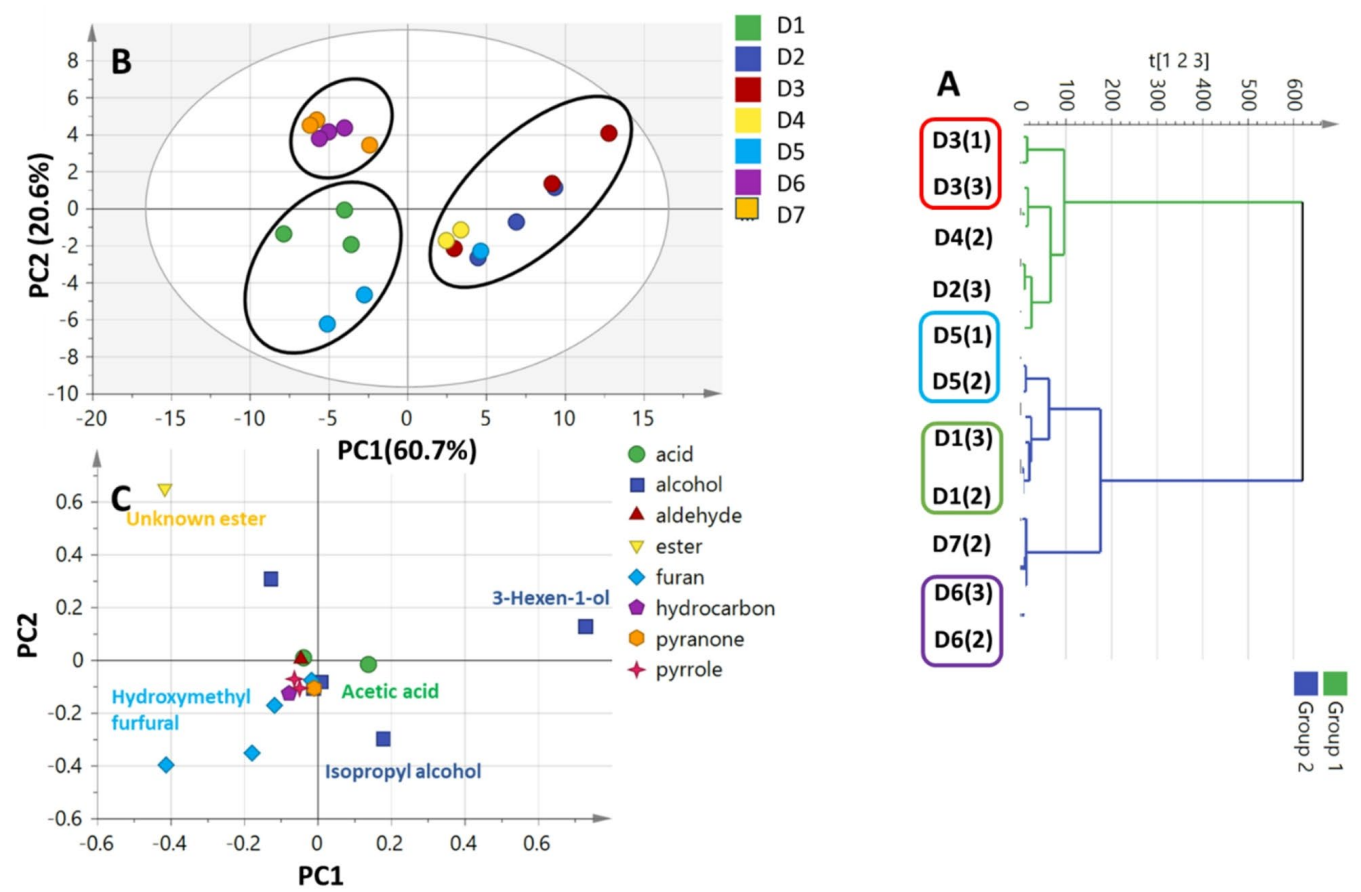
GC-MS based HCA and PCA of volatile metabolites from date syrup samples (*n* = 3). (A) HCA plot. (B) Score plot of PC1 vs. PC2 scores. (C) Loading plot for PC1 & PC2 contributing metabolites and their assignments. The metabolome clusters are located at distinct positions in two-dimensional space described by two vectors of principal component 1 (PC1) = 60.7% and PC2 = 20.6%.

To compare the classification power of nutrients in date syrup viz. sugars likely to serve as precursors for date syrup aroma, it was of interest to classify date syrup based on its aroma profile. HCA and PCA (Fig. 5) were employed to assess heterogeneity in the volatile distribution of date syrup products. The HCA dendrogram (Fig. 5A) revealed two separate clusters, with syrup products (D2, D3 & D4) clustered in group 1 versus (D1, D6 & D7) in group 2. The PCA score plot of the date syrup aroma profile dataset (Fig. 5B) accounted for 81% of the total variance. The segregation of D6 and D7 in one cluster on the upper left side and D1 and D5 in another cluster on the lower left side was demonstrated by the PCA model. The loading plot (Fig. 5C) revealed that alcohols (3-hexen-1-ol and isopropyl alcohol), furans (viz.5-hydroxymethyl furfural), and esters accounted for such segregation. Briefly, 5-hydroxymethyl furfural and unknown ester were enriched in D1, D5, D6, and D7. Whereas the highest levels of acetic acid, isopropyl alcohol, 3-hexen-1-ol accounted for D2, D3 & D4 clustering. Compared to the aroma dataset model, the primary metabolites offered a more reliable model for distinguishing between date syrup products. The distinct clustering determined by di-sugars richness emphasizes how crucial carbohydrate profiles are for distinguishing between different products. Future research should explore a combined approach that integrates chemical and sensory analysis to improve distinguishing between syrup products and understanding their distinctive qualities for better consumer appeal.

### UHPLC-MS metabolites profiling of date syrup

**Fig. 6:**
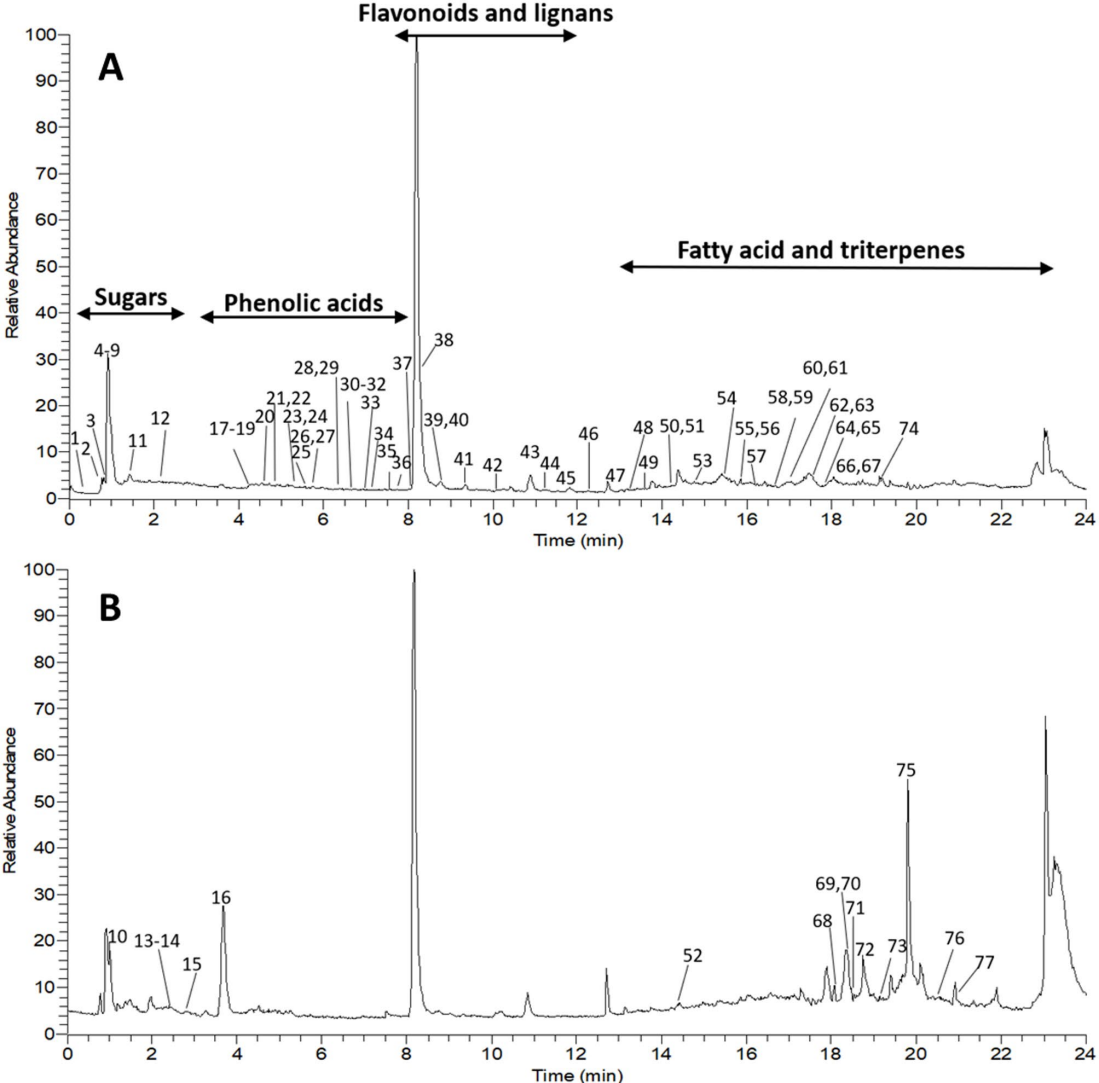
Representative total ion chromatogram of date syrup samples in negative (**A**) and positive (**B**) ionization mode.

Compared to GC-MS efficiency in primary metabolites profiling, UHPLC-MS was adopted to profile more secondary metabolites to likely account for date syrup health benefits. A total of 77 chromatographic peaks were annotated using UHPLC-MS (Fig. 6) of which 33 peaks are reported for the first time in *Phoenix dactylifera* (Table 2). Metabolites belonged to various metabolite classes including carbohydrates (10), phenolic acids (15), flavonoids (4), lignans (6), fatty acids (17), and terpenoids (5), as listed in Table 2.

**Table 2:** Metabolites identified in dates syrups analyzed via UHPLC-PDA-ESI-QToF/MS in negative and positive ionization modes.

Peak No.	RT	Metabolite name	Elemental composition	Mol. Ion m/z (-)(+)	Error	Fragments	References
1	0.47	Quinic acid	C_7_H_11_O_6_^−^	191.05511	2.5	163, 149, 147, 119, 85	^71^
2	0.85	Sorbitol	C_6_H_13_O_6_^−^	181.07159	0.5	119, 103, 101, 71	^51^
3	0.91	Trisaccharide*	C_19_H_33_O_18_^−^	549.1678	2.02	503, 341, 179, 161	^53^
4	0.93	Raffinose*	C_18_H_31_O_16_^−^	503.1624	2.37	383, 341, 179	^51^
5	0.96	Disaccharide	C_21_H_15_O_7_^−^	379.1564	3.5	377, 341, 179, 161, 143, 101, 89, 71	^51^
6	0.98	Disaccharide*	C_13_H_23_O_13_^−^	387.1147	2.5	221, 341, 179, 161, 143, 89, 71	^72^
7	1.04	Disaccharide*	C_18_H_17_O_9_^−^	377.0858	-3.86	341, 179	51
8	1.06	Sucrose	C_12_ H_21_O_11_^−^	341.10968	-2.35	179, 161, 89, 71	^55^
9	1.07	Hexose	C_6_H_11_O_6_^−^	179.05533	1.5	161, 131, 119, 101, 89, 71	^52^
10	1.08	Anhydro-di-hexose*	C_12_ H_21_O_10_^+^	325.1122	2.5	179, 109, 91	^73^
11	1.37	Citric acid	C_6_ H_7_ O_7_^−^	191.0193	3.5	173, 147	^74^
12	2.15	Dihexosyl glyceric acid*	C_15_H_25_O_14_^−^	429.1256	3.5	383, 323, 267, 179	
13	2.5	Cinnamic acid derivative*	C_14_ H_15_O_8_^+^	311.07352	7.5	295, 149	
14	2.58	Benzenetriol hexoside*	C_12_H_17_O_8_^+^	289.09204	4.5	271, 241, 211, 109, 81	
15	2.61	Benzenetriol*	C_6_H_7_O_3_^+^	127.03902	3.5	109, 81	^75^
16	3.66	Unidentified	C_14_H_15_O_8_^+^	311.07367	7.5	267, 185, 149, 109, 81	
17	4.28	Hydroxyferulic acid	C_10_H_9_O_5_^−^	209.04573	6.5	193, 179, 163	^55^
18	4.3	Protocatechuic acid-*O*- hexoside	C_13_H_15_O_9_^−^	315.0732	6.5	209, 181, 153, 109	^76^
19	4.42	Hydroxybenzoic acid-*O*-hexosyl pentoside*	C_18_H_23_O_12_^−^	431.12177	8.5	137, 93	^77^
20	4.73	Syringic acid-*O*-hexoside	C_15_H_19_O_10_^−^	359.09839	6.5	340, 197, 153	^74^
21	4.94	Piscidic acid	C_11_H_11_O_7_^−^	255.05086	1.5	165, 147, 107, 73	^78^
22	4.97	Caffeic acid-*O*-hexoside	C_15_H_17_O_9_^−^	341.08746	7.5	179, 135	^58^
23	5.14	*O*-Dicaffeoyl shikimic acid	C_22_H_25_O_13_^−^	497.1316	4.19	335, 179, 161	^58^
24	5.22	Hydroxy benzoic acid	C_7_H_5_O_3_^−^	137.02422	5.5	93	^55^
25	5.64	Acyl sucrose	C_17_H_29_O_12_^−^	425.16736	3.5	381, 241	55
26	5.78	Ferulic acid-*O*-dihexoside	C_22_H_29_O_14_^−^	517.15692	8.5	324, 193, 175	^55^
27	5.8	Coumaric acid-*O*-hexoside	C_15_H_17_O_8_^−^	325.09509	7.5	163, 119	^58^
28	6.34	Benzoic acid*	C_7_H_5_O_2_^−^	121.02916	5.5	93, 75	^79^
29	6.39	Ferulic acid-*O*-hexoside	C_16_H_19_O_9_^−^	355.1045	7.5	193, 149	^55^
30	6.6	Caffeoyl-*O*-shikimic acid	C_16_H_15_O_8_^−^	335.0785	5.3	161, 156	^55^
31	6.69	Benzylalcohol-*O*-apiosyl hexoside*	C_19_H_27_O_12_^−^	447.152	6.5	269	^80^
32	6.71	Luteolin-*O*-rhamnosyl hexoside	C_27_H_29_O_15_^−^	593.15479	6.99	285	^55^
33	6.85	Benzyl-*O*-hexosyl pentoside*	C_18_H_25_O_10_^−^	401.0883	6.5	269, 161	^81^
34	7.2	Unidentified	C_20_H_33_O_11_^−^	449.20206	4.5	403, 301	
35	7.58	Methylbutyl-*O*-pentosyl hexoside*	C_16_H_29_O_10_^−^	381.17706	2.5	294, 161, 101, 71	^82^
36	7.85	Dihydrocoumaric acid*	C_9_H_9_O_3_^−^	165.05521	5.5	163, 147, 119	^83^
37	8.13	Carboxymethoxy-methylcoumarin*	C_12_H_9_O_5_^−^	233.04517	8.5	215, 193, 175, 161	
38	8.31	Chrysoeriol-*O*-hexoside	C_22_H_21_O_11_^−^	461.10968	2.8	299, 254, 161, 149	^84^
39	8.7	Lignan derivative*	C_29_H_39_O_15_^−^	627.2313	3.5	581, 419, 373	^63^
40	8.81	Lignan derivative*	C_35_H_47_O_20_^−^	787.269	3.7	417, 369, 317	
41	9.35	*p*-Coumaric acid	C_9_H_7_O_3_^−^	163.03952	6.5	119	^59^
42	10.06	Lyoniresinol-*O*-hexoside	C_28_H_37_O_13_^−^	581.2254	-2.63	419, 389, 371	63
43	10.89	Syringaresinol-*O*-hexoside*	C_28_H_35_O_13_^−^	579.2099	3.6	548, 417, 402, 387, 312	^62^
44	11.21	Chrysoeriol-*O*-hexosyl sulfate	C_22_H_21_O_14_S^−^	541.06671	2.79	417, 339, 299, 284, 265, 241	^19^
45	11.73	Syringaresinol*	C_22_H_25_O_8_^−^	417.15375	-2.86	287, 373	62
46	12.25	Chrysoeriol-*O*-rhamnosyl hexoside	C_28_H_31_O_15_^−^	607.16846	3.57	299, 284	19
47	12.96	Unidentified	C_15_H_16_O_9_^−^	340.0834	8	322, 294	
48	13.3	Abscisic acid	C_15_H_19_O_4_^−^	263.1291	6.5	248, 219	^85^
49	13.62	Secoisolariciresinol	C_20_H_25_O_6_^−^	361.16647	8.5	346, 333	^64^
50	14.21	Unidentified	C_14_H_11_O_5_^−^	259.06094	1.13	231, 185	
51	14.24	Trihydroxy octadecadienoic acid	C_18_H_31_O_5_^−^	327.21869	3.5	211, 171	^58^
52	14.37	Hydroxy octadecatrienoic acid	C_18_H_31_O_3_^+^	295.2269	3.5	277, 249, 73	^86^
53	14.76	Trihydroxy oleic acid	C_18_ H_33_O_5_^−^	329.23428	2.5	311, 293, 211, 171	^55^
54	15.34	Di-furanyl dimethylundecatrien-ol*	C_21_H_25_O_3_^−^	325.18521	9.5	279, 183	^87^
55	15.82	Dihydroxy oleic acid	C_18_H_33_O_4_^−^	313.23953	2.5	295, 183	^88^
56	15.9	Di-furanyl trimethylundecatrien-ol*	C_22_H_27_O_3_^−^	339.20056	7.5	295, 277, 197, 183	
57	16.25	Dihydroxy octadecanoic acid	C_18_H_35_O_4_^−^	315.25488	1.5	297, 279	^88^
58	16.68	Unidentified	C_22_H_27_O_4_^−^	355.19556	9.5	337, 297, 241, 199	
59	16.73	Hydroxy-oxo-octadecadienoic*	C_18_H_29_O_4_^−^	309.20789	4.5	291, 265, 183	^89^
60	17.09	Linoleic acid	C_18_H_31_O_2_^−^	279.23282	3.5	183	^88^
61	17.19	Hydroxy octadecatrienoic acid isomer	C_18_H_29_O_3_^−^	293.21271	4.5	185, 125, 102	^90^
62	17.37	Hydroxy-oxo-octadecatetranoic acid*	C_18_H_25_O_4_^−^	305.17645	6.5	261, 155, 134, 121	90
63	17.58	Hydroxy stearic acid	C_18_H_35_O_3_^−^	299.2598	2	183	88
64	17.91	Hydroxy octadecadienoic acid	C_18_H_31_O_3_^−^	295.22873	3.5	251, 185	88
65	17.99	Hydroxy palmitic acid	C_16_H_31_O_3_^−^	271.22787	1.5	225	^59^
66	18.16	Oleic acid	C_18_H_33_O_2_^−^	281.24887	2.5	209, 181	^88^
67	18.25	Ursolic acid	C_30_H_47_O_3_^−^	455.35458	7.5	441, 346, 327, 240, 219	90
68	18.28	Unknown triterpenoid*	C_30_H_49_O_7_^+^	521.34503	6.5	497, 451, 375, 257	
69	18.35	Unknown fatty acid*	C_19_H_37_O_3_^+^	313.27365	1.5	269	
70	18.38	Stearate derivative*	C_25_H_47_O_6_^+^	443.33447	2.5	284, 261, 232, 207	
71	18.4	Linoleoyl glycerol*	C_21_H_39_O_4_^+^	355.28183	2.5	285, 91, 73	^91^
72	18.71	Unknown triterpenoid*	C_30_H_57_O_8_^+^	545.4024	2.5	474, 390	
73	19.12	Unknown triterpenoid Oleane triterpene*	C_30_H_49_O_6_^+^	505.35019	6.5	473, 451, 284	
74	19.25	Estrone acetate	C_20_H_23_O_3_^−^	311.16959	9.5	183	^55^
75	19.82	Unknown fatty acid*	C_24_H_45_O_4_^+^	397.32889	2.5	335, 271	
76	20.49	Unknown triterpenoid*	C_30_H_57_O_6_^+^	513.41254	2.5	423, 345, 299, 265	
77	20.88	Stearate derivative*	C_25_H_47_O_6_^+^	443.33447	2.5	284, 261	

* First detected in *Phoenix dactylifera*.

### Sugars

Sugars, mainly reducing sugars, are considered the main component of dates^48^and to account for 73% of date’s dry weight^49^. UHPLC coupled with MS is currently a powerful analytical tool for sugar analysis^50^. For the monosaccharide of hexose structure, molecular ion at *m/z* 179 [M − H]^−^ showed further masses at *m/z* 161 and 143 due to the subsequent loss of two water moieties^51^. Peak 9 with a molecular ion peak at *m/z* 179.055 was assigned as hexose (glucose/ fructose) a major sugar in dates^52^. Trisaccharides (peaks 3 & 4) and disaccharides (peaks 5, 6, 7, 8 & 25) showed fragment ions at *m/z* 341 and 179, respectively due to neutral loss of hexose moiety^51,53^. Peak 8 yielded fragment ions at *m/z* 179 and 161 corresponding to [M–H–C_6_H_12_O_6_]^−^ and [M–H–C_6_H_12_O_6_–H_2_O] ^−^ and annotated as sucrose which produced due to low invertase activity^54^. Peak 25 was annotated as acyl sucrose as previously reported^55^. As with most cultivars of date fruits^56^date syrup samples exhibited lower levels of di- and tri- sugars compared to mono-sugars.

### Phenolic acids

Several studies have reported on date richness in phenolic acids belonging to cinnamates and benzoates^49^accounting for fruit taste and health benefits. Date fruits are rich in phenolics i.e., caffeic, ferulic, protocatechuic, catechin, gallic, *p*-coumaric, resorcinol, chlorogenic, and syringic acids, that vary depending on the plant’s origin, variety, extraction process, and measuring technique^57^. In the present work, a total of 15 phenolic conjugates were detected showing typical neutral losses of H_2_O [M − H−18]^−^ and CO_2_ [M − H−44]^−^ characteristic of phenolic acids or their derivatives. A base peak at *m/z* 179 [M − H−162]^−^ due to the loss of hexose in peak 22 led to its assignment as caffeic acid-*O*-hexoside. Likewise, peaks 23 and 30 showed fragment ion at *m/z* 179 diagnostic for caffeic acid led to their identification as dicaffeoyl-*O*-shikimic acid and caffeoyl-*O*-shikimic acid, respectively^19,55^. Peak 29 was annotated as ferulic acid-*O*-hexoside based on product ion at *m/z* 193 (deprotonated ferulic acid) post cleavage of a hexose moiety. Additional ferulic acid conjugates were assigned as hydroxyferulic acid (peak 17) and ferulic acid-*O*-dihexoside (peak 26) showing aglycone moiety of ferulic acid at *m/z* 19^55^ and aiding in identification. Other cinnamates were detected in peaks 27, 36 and 41, showing base peak at *m/z* 119 characteristic for *p*-coumaric conjugates, and were annotated as *p*-coumaric acid-*O*-hexoside, dihydrocoumaric acid and *p*-coumaric acid, respectively^58,59^.

Aside from cinnamates, benzoates were also detected in date syrup samples (peaks 18, 19, 20, 24, 28, 31 and 33). MS/MS spectra of peak 18 showed a base peak at *m/z* 153 due to the loss of hexose, fragment ion at *m/z* 109 characteristic for protocatechuic acid and annotated as protocatechuic acid-*O*-hexoside^60^. Peak 20 was assigned as syringic acid-*O*-hexoside with MS^2^ fragments at *m/z* 197 [M–H–162]^−^ and 153 post loss of CO_2_. Other identified benzoates included hydroxy benzoic acid (24) and benzoic acid (28)^55^. It is worth noting that cinnamate derivates were more prevalent than benzoates in date syrup samples under investigation.

### Flavonoids and lignans

Flavonoids from different sub-classes including flavonols, flavones, and flavanones glycosides of quercetin, naringenin, myricetin, apigenin, kaempferol, and luteolin have been reported from date fruit^19,55,61^. Likewise, 4 flavonoids (peaks 32, 38, 44 and 46) belonging to flavones were observed in date syrup samples, with chrysoeriol derivatives as major forms. Chrysoeriol-*O*-rhamnosyl hexoside (peak 46) was characterized at *m/z* 607.16846 with fragment ions of chrysoeriol aglycone at *m/z* 299 and 284 aiding in its identification^19^. Other chrysoeriol glycosides included chrysoeriol-*O*-hexoside and chrysoeriol-*O*-hexosyl sulfate in peaks 38 and 44, respectively with prominent fragment ions at *m/z* 299. Another flavonoid di-glycoside was assigned as luteolin-*O*-rhamnosyl hexoside (peak 32) at *m/z* 593.15479 and yielded fragment ion at *m/z* 285 (aglycon ion) post sugar moieties cleavage^55^and suggestive that flavones represent major flavonoid subclass in date syrup.

With regards to lignans, peak 45 at *m/z* 417.15375 (C_22_H_25_O_8_^−^) and peak 43 at *m/z* 579.2099 (C_28_H_35_O_13_^−^) were annotated as syringaresinol and syringaresinol-*O*-hexoside, respectively, based on their molecular formula and tandem MS data^62^. Moreover, peak 42 was annotated as lyoniresinol-*O*-hexoside with fragments at *m/z* 419 and 389 due to lose of hexose and -OCH_2_ moieties, respectively^63^. Peak 49 at *m/z* 346.141 (C_20_H_25_O_6_^−^) was annotated as secoisolariciresinol^64^ showing fragment ion *m/z* 346 after the loss of methyl radical. In date syrup samples, lignans were found to be more prevalent than flavonoids based on peak area.

#### Fatty acids

In the second half of the UHPLC/MS chromatogram, several fatty acids were observed showing late elution considering their nonpolar nature with hydroxy fatty acids mostly predominated. For instance, peaks 52, 63 and 65 were annotated as hydroxy linolenic acid, hydroxy stearic acid and hydroxy palmitic acid, respectively. Moreover, peaks 53 and 55 at *m/z* 313.23953 and 329.23428 were annotated as dihydroxy oleic acid and trihydroxy oleic acid, respectively. Similarly, peaks 55, 57, 59 and 62 were identified as dihydroxy octadecenoic, dihydroxy octadecanoic, hydroxy-oxo-octadecadienoic and hydroxy-oxo-octadecatetranoic acids respectively. Even though sphingolipids were previously detected in various date parts and products^19,55^ none were detected in the investigated date syrup samples.

#### Steroids

Estrone acetate was identified in peak (74) showing fragment ion at *m/z* 183 due to the successive losses of CH_3_CO and C_5_H_10_O which was previously reported in date palm pollen^55^. It has also been reported that estrone is present in palm pollens which can be suggestive for the possible admixture during collection^65^.

### Multivariate PCA, HCA and OPLS-DA analyses of UHPLC–MS dataset

#### PCA and HCA multivariate analysis of UHPLC–MS data

**Fig. 7:**
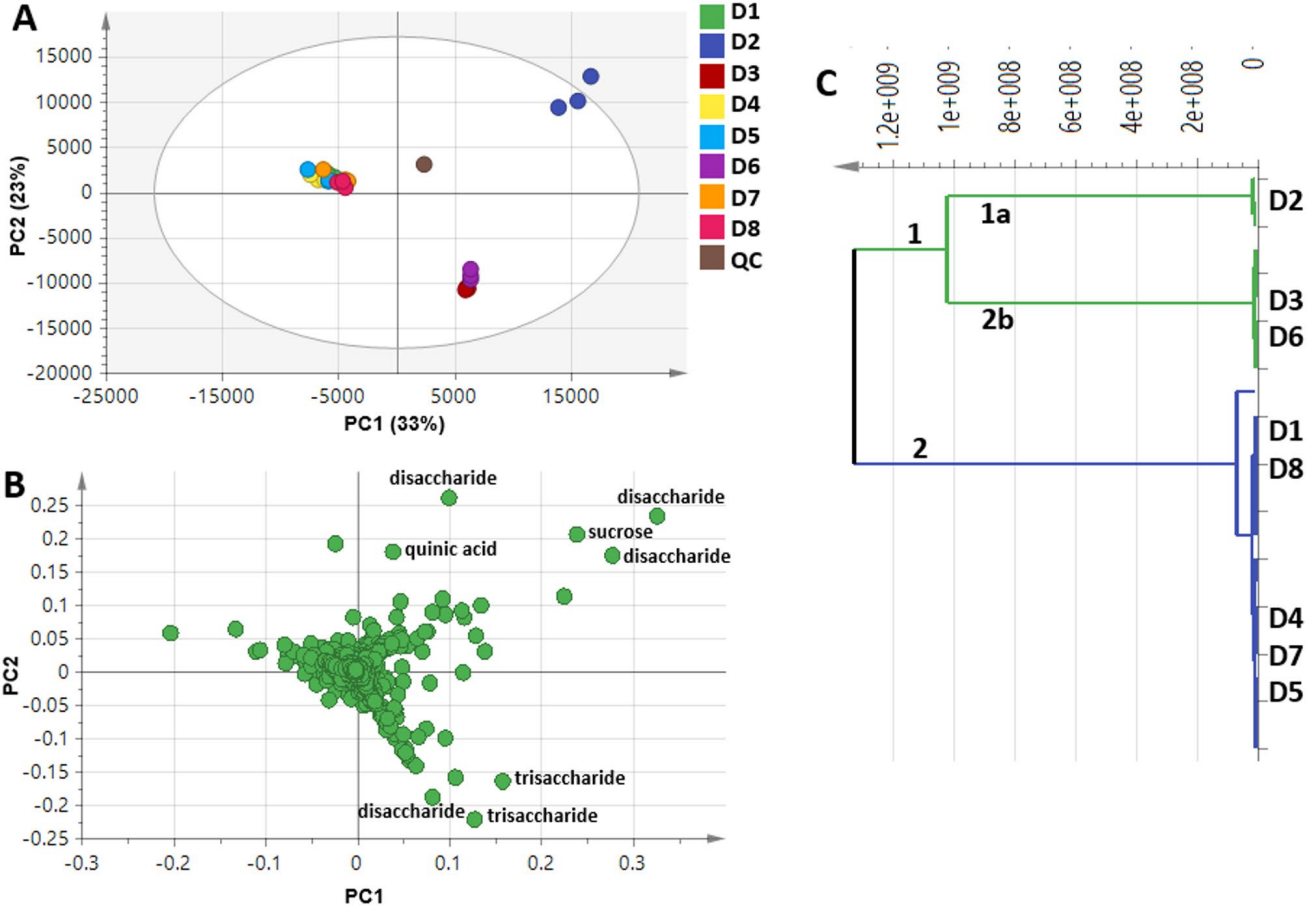
LC/MS-based unsupervised multivariate data analyses of whole sample dataset under investigation. (**A**) PCA score plot of PC1 versus PC2 scores. (**B**) PCA loading plot for PC1 and PC2 contributing metabolites and their assignments. (*A*) HCA plot.

To assess metabolites heterogeneity among data syrups, unsupervised PCA) and hierarchical and HCA were performed. PCA was prescribed by two orthogonal components, accounting for 56% of the total variance, with distinct segregation of samples D2, D3 and D6 at the right side of PC1, while almost all other samples were positioned on the left side of the plot (negative PC1 values) (Fig. 7A). The model explains 56% of the total variance (R2 = 0.56), with positive prediction goodness parameter (Q2 = 0.41) suggesting the validity of the model^66^ (Fig. S3).

The loading plot (Fig. 7B) revealed that sugars contributed the most in samples discrimination. Disaccharides (peaks 5, 6, 7 and 8) and trisaccharides (peaks 3 and 4) were found enriched in samples D2, D3 and D6. Such a clustering pattern was also revealed in HCA which showed two distinct major groups of 3 and 5 samples (Fig. 7C). Examination of the dendrogram showed that samples D2, D3 and D6 were the most distant ones, and were clustered in two subgroups as D2 (cluster 1a) is clearly segregated from D3 and D6 (cluster 1b), and in agreement with PCA results.

Further, PCA and HCA (Fig. S4C) models were employed to classify date syrup samples with regards to secondary metabolites after excluding sugar peaks. PCA failed to discriminate among samples, with total variance coverage of 27% (Fig. S4A). This value revealed that variance between samples is low and needs more dimensions to cover all aspects^67^. Investigation of the loading plot (Fig. S4B) revealed that furans i.e. di-furanyldimethylundecatrien-ol (peak 54) and di-furanyltrimethylundecatrien-ol (peaks 56), which could result from sugar oxidation during syrup manufacturing process, accounted for the segregation of samples along PC1.

#### OPLS-DA multivariate supervised analysis of UHPLC–MS data

**Fig. 8:**
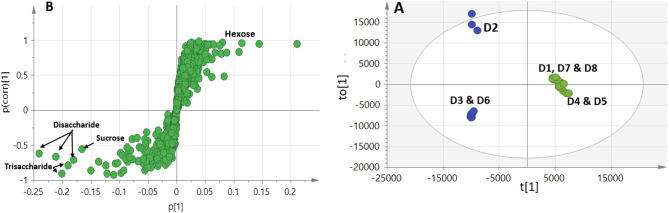
LC/MS-based OPLS analysis of metabolites analyzed in whole date syrup samples. (**A**) OPLS-DA score plot (*n* = 3). The respective loading S-plots (**B**) show the covariance p[1] against the correlation p(cor)[1] of the variables of the discriminating component of the OPLS-DA model.

OPLS-DA supervised modeling was implemented to confirm results derived from PCA and HCA results regarding date syrup differences in metabolome (Fig. 8).

The model showed predictability Q2 = 98.5%, total variance coverage R2 = 99.2% and *p*- value of less than 0.05. The corresponding S-plot revealed that di- and tri-sugars were enriched in samples D2, D3 and D6, while monosaccharides (i.e. hexose) were enriched in the other samples, and in agreement with PCA loading plot results. Notably, phenolics, flavonoids and fatty acids detected in all samples did not contribute for segregation in PCA or OPLS results. Results from PCA, HCA, and OPLS-DA fall in agreement with another report showing that sugars were the predominant metabolite class in the date syrup samples using UHPLC-MS ^48^. The primary metabolites derived models of both GC-MS and UHPLC–MS demonstrated superior performance in classifying date syrup products compared to the aroma profile classification and the secondary metabolites UHPLC–MS models. Although our data suggest that factors beyond geographic origin contribute to the observed chemical variation, the limited number of samples, especially being of the same batches imposes constraints on the interpretation. With regards to date syrup acquisition of different origins, with only two samples available from Saudi Arabia, it was not possible to establish a statistically valid conclusion on how geographic origin influences the chemical profile of the date syrups.

### Total phenolics and flavonoids assays of date syrup samples

Date fruit is rich in phenolic compounds with differences depending on variety type and extraction methods^68^. The enrichment in phenolics in date syrup samples as revealed via UHPLC/MS analysis (Table 2) prompted assays for determination of total phenolics and flavonoids for standardization purposes, especially since UHPLC/MS models failed to discriminate samples based on phenolics. Considering that D7 did not demonstrate any distinguishing features as a variant in GC-MS or UHPLC–MS data models, proximate assay was performed for D1-D6. Results (Table S3) revealed that D1 exhibited the highest phenolic content (258.083 ± 4.93 mg GAE/g) versus the lowest in D2 (94.944 ± 2.33 mg GAE/g). Total flavonoids showed the same pattern in D1 and D2 respectively at 239.888 ± 6.81 and 53.920 ± 3.41 mg QE⁄g, respectively. This is aligned with findings regarding date fruit that showed high total phenolics and flavonoids considering cultivar type and ripening stage. Importantly, D1 showed higher phenolic and flavonoid content than some date varieties^49^. Such results pose date syrup as a potential source of phenolics, in agreement with previous reports^69^and warranting for assessment of its antioxidant effects.

### In vitro antioxidant activity of date syrups via DPPH radical scavenging assay

Date fruit exhibits good antioxidant activity attributed to its antioxidants i.e. phenolic acids and flavonoids^57,70^ and likewise expected in date syrup samples in this study. The DPPH assay was used as a rapid and sensitive method for the evaluation of free radical scavenging ability of date syrup samples. The results are expressed as IC_50_ values (Fig. S5). All samples exhibited dose-dependent radical scavenging effects, with D1 exhibiting the strongest antioxidant activity (IC_50_ = 88.20 ± 5.82 µg/mL) followed by D5 (IC_50_ = 110.64 ± 8.50 µg/mL) when compared to gallic acid (IC_50_ = 21.55 ± 3.06 µg/ mL). The higher antioxidant potential of D1 and D2 syrups are attributed to their relatively high total phenolic and flavonoid content (Table S3). Clearly, all samples exhibited antioxidant activity that was less than that of gallic acid. Pearson correlation showed a significant negative correlation between total phenolic content and DPPH radical scavenging activity (*r* = − 0.9822, *p* < 0.05), as well as between total flavonoid content and DPPH activity (*r* = − 0.9123, *p* < 0.05), indicating that flavonoids and phenolics play a significant role in contributing to the antioxidant properties of the analyzed syrups. Further research is recommended to explore the mechanisms of such relationship and its implications for food quality and health benefits.

## Conclusion

A UHPLC-MS and GC-MS metabolomics approach was utilized to compare the chemical composition of commercial date syrup of different origins in Egypt and Saudi Arabia as major producers of date syrup. Mono-sugars predominated all products, especially fructose, except D2 which showed high sucrose levels. Such a high concentration of simple sugars makes date syrup as an instant energy source like date fruit. All examined products appeared suitable for diabetic patients except D2 due to their high fructose and mannitol content, which has yet to be confirmed by measuring the increase in post-prandial sugar levels post-consumption. Many factors could contribute to the observed variation in sugar profiles among examined date products e.g., dates’ cultivar, degree of maturation, processing conditions, and /or addition of sweeteners during preparation. Hence at least *cvs* and stage of maturity of the utilized date fruits should be stated on the product label. The sugar profile poses syrup D3 and D6 as the best-balanced products in sweetness due to their higher maltose and allose, concurrent with lower fructose levels. GC-MS was further utilized to profile aroma belonging mainly to furans, alcohols, and esters, with furans derived from the processing step of sugars conversion. The syrup aroma is not considered as rich as in date fruit, where several compounds disappeared especially terpene hydrocarbons, oxygenated monoterpenes, benzenoids, in addition to many esters, aldehydes, alcohols, and acids. Future studies should compare the different processing methods utilized during syrup preparation (e.g., thermal, hydraulic pressure, microwave, ultrasound, and ohmic heating) in the context of the syrup metabolite profile to identify the best methods in preparation.

Both UHPLC-MS and GC-MS analyses confirm that sugars are the major components of date syrup. The classification of the UHPLC-MS dataset was influenced by variations in sugar composition, which were also observed in GC-MS results. While both techniques effectively differentiated samples D2, D3, and D6 attributable to differences in disaccharide and trisaccharide content, GC-MS further enabled segregation based on monosaccharide profiles. This suggests that GC-MS provides a more robust model for the classification of date syrup products. This study provided the first comprehensive metabolic profiling of date syrups, emphasizing their nutritive value and potential health benefits.

## Supplementary Information

Below is the link to the electronic supplementary material.

Supplementary Material 1

## Data Availability

The datasets used and/or analyzed during the current study can be made available from the corresponding author on reasonable request.
