# Supplementary material for: Metabolomic and antioxidant characterization of seven Egyptian and Saudi date syrups via GC-MS and UHPLC/MS with sensory bioactive insights
Source: Sci Rep. 2025 Sep 18;15:32604. doi: 10.1038/s41598-025-19541-2 (PMC12446427; doi:10.1038/s41598-025-19541-2)
Supplement: Supplementary file 1 — Supplementary Material 1 [file 41598_2025_19541_MOESM1_ESM.pdf]

Supplementary Material

Metabolomic and antioxidant characterization of seven Egyptian and Saudi date syrups via GC-MS and UHPLC/MS with sensory bioactive insights

Rabab M. Abdou[1a], Heba Fahmy[1b], Amira R. Khattab[c,d], Mohamed A. Farag[a,e*]

[1] Equal contribution

[a] Pharmacognosy Department, Faculty of Pharmacy, Cairo University, Cairo, Egypt
[b] Pharmacognosy Department, Faculty of Pharmacy, Modern University for Technology & Information, Cairo, Egypt
[c] Pharmacognosy Department, College of Pharmacy, Arab Academy for Science, Technology and Maritime Transport, Alexandria, 1029, Egypt

[d] Graduate School in Alamein, Arab Academy for Science, Technology and Maritime Transport, Alamein, Egypt.

[e]Healthcare faculty, Saxony Egypt University (SEU), Badr City, Egypt.

*Corresponding author at: Pharmacognosy Department, College of Pharmacy, Cairo University, Cairo, Egypt, Kasr El Aini St, P.B, 11562, Egypt. E-mail address: mohamed.farag@pharma.cu.edu.eg

Table S1. Sample codes for date syrup commercial samples used in the study

Sample Codes	Company name	Production Country
**D1**	Abo ouf	Egypt
**D2**	Watania Organic	Saudi Arabia
**D3**	dar el Tayseer	Siwa-Egypt
**D4**	El nakhiel	Saudi Arabia
**D5**	Imtenan Group	Obour city, Egypt
**D6**	Araab	Egypt
**D7**	Tahan	Egypt

Table S2. Relative percentage of volatile metabolites in date syrups analyzed via GC–MS, n = 3. For codes, refer to Table S1. Results are represented as an average of 3 independent replicates ± std. deviation.

Peak	Rt (min)	KI	Metabolite name		D1	D2	D3	D4	D5	D6	D7
**Acids**											
1	0.858	520	Acetic acid	Acid	1.48±1.41	3.04±0.37	2.47±1.34	1.50±0.64	1.54±1.24	0.83±0.12	0.89±0.45
2	4.501	1135	Butanoic acid	Acid	0.25±0.04	0.13±0.04	0.29±0.13	0.41±0.23	0.29±0.12	0.43±0.06	0.41±0.17
**Total acids**					**1.73**	**3.17**	**2.76**	**1.91**	**1.83**	**1.26**	**1.31**
3	1.338	598	Isopropyl Alcohol	Alcohol	2.59±1.14	10.23±4.97	6.41±1.61	8.13±2.56	7.53±1.98	1.98±0.07	2.50±0.22
4	1.556	632	3-Hexen-1-ol	Alcohol	11.85±7.10	48.28±12.49	58.74±18.60	28.00±13.99	22.91±16.32	14.78±3.21	14.79±7.19
5	1.657	649	4-Penten-2-ol	Alcohol	1.18±0.34	1.68±0.44	1.80±0.29	4.53±0.83	1.79±0.61	7.77±0.65	7.57±1.71
6	2.333	759	Unknown	Alcohol	0.65±0.11	0.51±0.24	0.37±0.07	0.74±0.19	0.61±0.15	0.27±0.10	0.28±0.10
7	3.624	968	α-Terpineol	alcohol	0.35±0.33	0.74±0.57	0.39±0.18	0.22±0.10	0.51±0.25	0.12±0.07	0.18±0.01
**Total alcohols**					**16.62**	**61.43**	**67.71**	**41.63**	**33.36**	**24.92**	**25.33**
8	3.538	954	Unknown	ester	19.88±4.28	2.58±1.33	1.65±0.65	2.57±0.98	3.56±2.34	27.67±0.66	28.34±4.74
Total esters											
9	1.478	619	Furfural	furan	22.52±1.85	19.27±1.63	6.95±3.25	17.38±4.80	21.15±6.93	15.99±1.32	14.68±1.79
10	1.992	702	Furfural, 5-methyl-	furan	3.92±0.31	0.30±0.01	0.39±0.26	2.54±1.12	3.31±1.18	1.54±0.06	1.36±0.26
11	2.517	783	Methyl 3-furoate	furan	0.41±0.02	0.26±0.09	0.56±0.51	0.74±0.14	0.61±0.18	0.40±0.07	0.36±0.07
12	3.142	888	5-Hydroxymethylfurfural	furan	27.41±6.35	9.79±4.33	16.36±12.92	27.85±11.11	30.42±16.24	24.14±0.88	24.43±3.12
**Total furans**					**54.25**	**29.62**	**24.27**	**48.50**	**55.49**	**42.07**	**40.84**
13	1.781	668	2,6-Octadiene, (E,E)-	Hydrocar bon	2.80±0.13	0.50±0.09	0.61±0.26	1.29±0.38	2.03±0.95	0.81±0.10	0.97±0.22
14	2.692	816	Maltol	pyranone	0.64±0.15	0.56±0.03	0.37±0.16	1.17±0.57	0.49±0.22	0.29±0.06	0.32±0.11
15	2.45	777	2-Acetylpyrrole	pyrrole	1.39±0.20	0.60±0.00	0.34±0.15	0.87±0.42	0.93±0.32	0.45±0.19	0.49±0.21
16	2.524	789	N-Ethylpyrrole	pyrrole	1.92±0.57	1.35±0.04	1.60±1.25	1.68±0.79	1.45±0.35	1.48±0.26	1.64±
17	3.287	911	P-Anisaldehyde	Aldehyde	0.77±0.44	0.18±0.02	0.69±0.49	0.37±0.26	0.86±0.15	1.07±1.16	0.76±
				**Sum**	**100.00**	**100.00**	**100.00**	**100.00**	**100.00**	**100.00**	**100.00**

Table S3: Total phenolics and total flavonoids of date syrup samples (values expressed are means ± S.D. of three measurements)

Sample	TF mg QE⁄g	TPC (mg GAE/g)
**D1**	239.88±6.81	258.09±4.93
**D2**	53.92±3.41	94.94±2.33
**D3**	66.17±6.54	130.08±3.31
**D4**	170.55±5.96	207.53±3.47
**D5**	180.15±5.88	242.56±3.97
**D6**	114.07±3.86	128.29±3.41

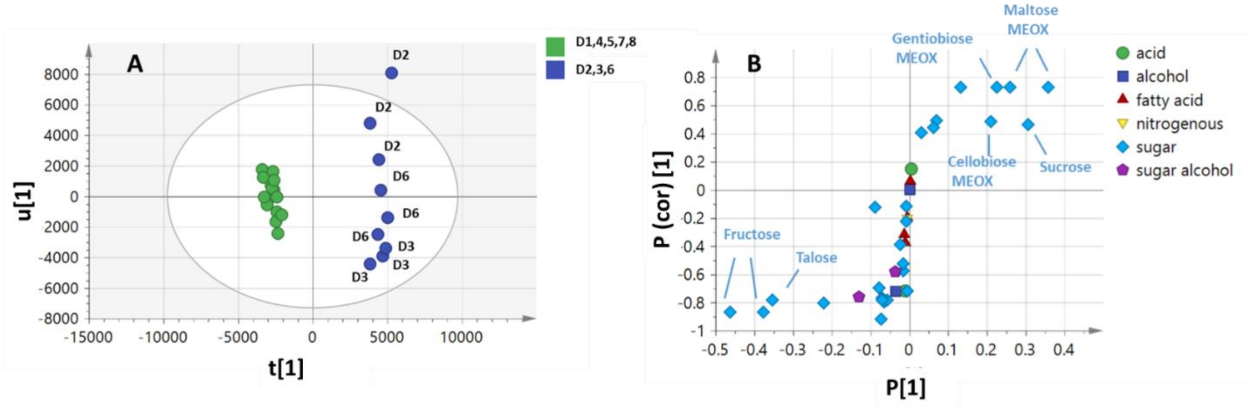

Fig. S1 GC-MS based OPLS-DA score plot derived from modelling of D2, D3, and D6 against other date syrup samples (n=3) (A). The respective S-plot (B) shows the covariance p [1] against the correlation p(cor)[1] of the variables of the discriminating component of the OPLS-DA model. Cut-off values of P<0.05 were used; selected variables are highlighted in the S-plot with identification.

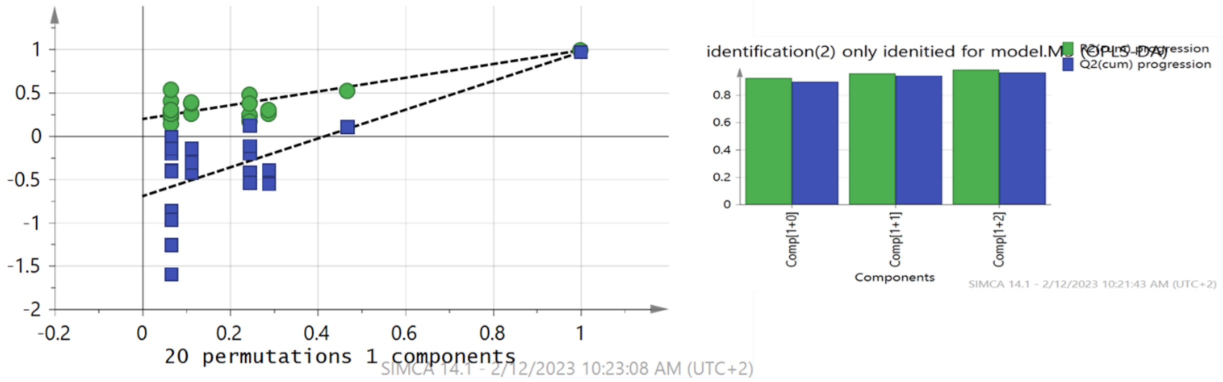

M3(Untitled)	SS	DF	MS	F	p	SD
Total corr.	23	23	1			1
Regression	22.2021	6	3.70034	78.8346	1.83058e-011	1.92363
Residual	0.797946	17	0.046938			0.216652

Fig. S2 Validation of model, permutation p-value statistically significant p<0.05

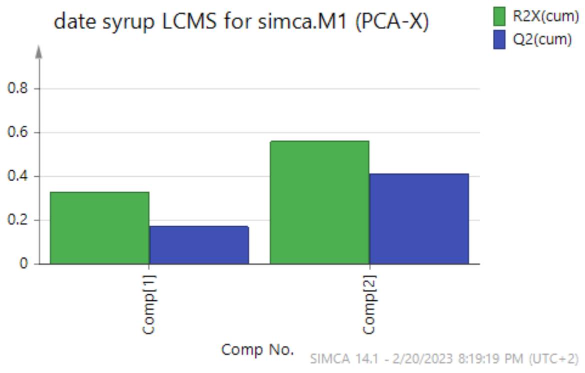

Comp No.	M1.R2X(cum)	M1.Q2(cum)
Comp[1]	0.333595	0.176114
Comp[2]	0.562396	0.413758

Fig. S3 Validation of LC-MS model

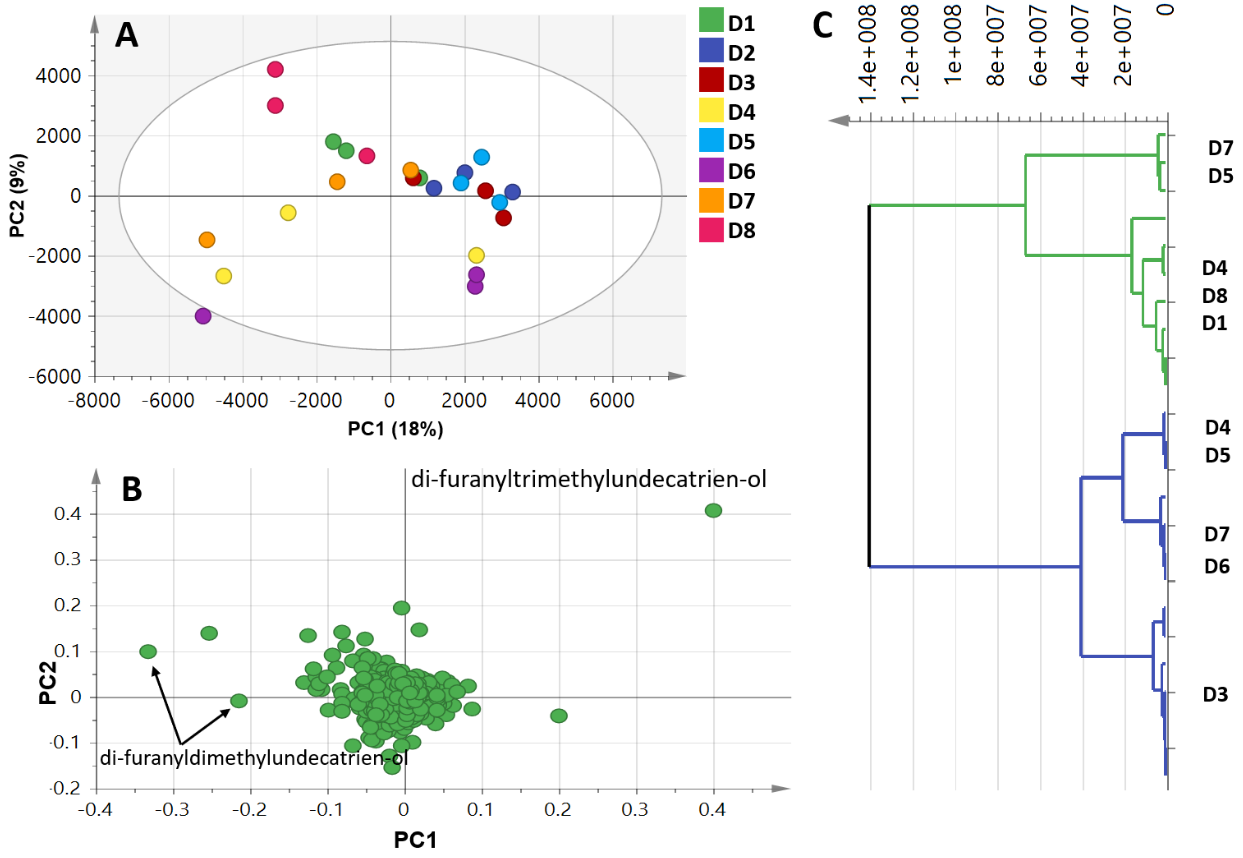

Fig. S4 UHPLC-MS-based unsupervised multivariate data analyses of whole sample dataset without sugar peaks. (A) PCA score plot of PC1 versus PC2 scores. (B) PCA loading plot for PC1 and PC2 contributing metabolites and their assignments. (A) HCA plot.

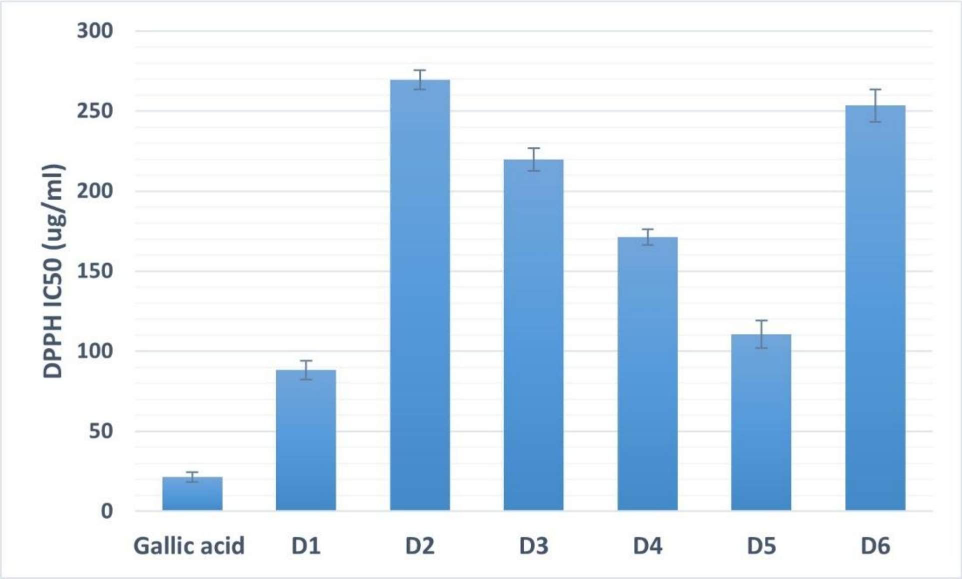

Fig. S5 DPPH IC50 of date syrup sample
